# Deep Learning Based Surface Classification of Functionalized Polymer Coatings

**DOI:** 10.1021/acs.langmuir.4c03971

**Published:** 2025-04-30

**Authors:** Safoura Vaez, Diba Shahbazi, Meike Koenig, Matthias Franzreb, Joerg Lahann

**Affiliations:** † Institute of Functional Interfaces (IFG), Karlsruhe Institute of Technology (KIT), Hermann-von-Helmholtz-Platz 1, 76344, Eggenstein-Leopoldshafen, Germany; ‡ Biointerfaces Institute, Departments of Chemical Engineering, Materials Science and Engineering, and Biomedical Engineering, and the Macromolecular Science and Engineering Program, University of Michigan, Ann Arbor, Michigan 48109, United States

## Abstract

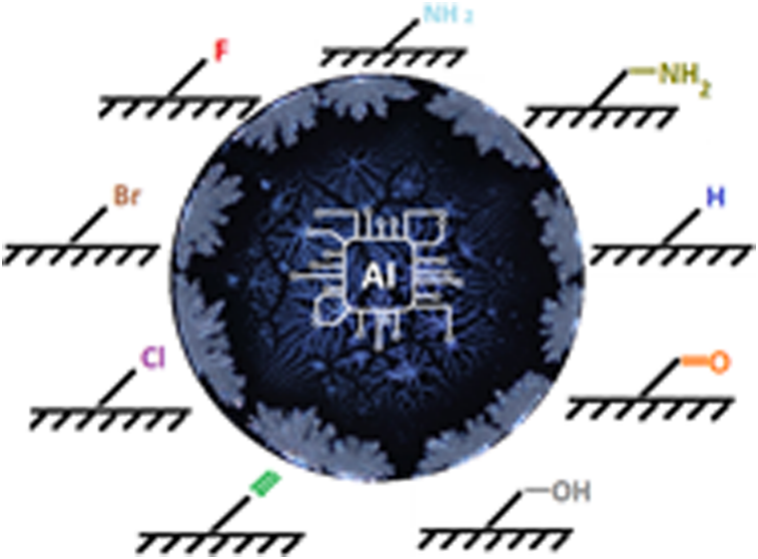

Low-technology characterization of material surfaces poses a challenge of significant importance for many scientific fields such as medical implants, biosensors, and regenerative medicine. Simple, fast, and scalable surface analysis methods that can be applied to a wide range of functionalized polymer coatings would thus constitute a major scientific and technological advance. In this work, we studied stain patterns formed by depositing a defined protein solution onto various polymer surfaces. The images of the resulting drying droplet patterns were captured by polarized light microscopy and analyzed by a deep-learning neural network. In this proof-of-concept study, we used chemical vapor deposition polymerization to deposit ten structurally distinct polymer coatings that share an identical polymer backbone, but differ in their functional groups. Despite the relatively minute differences in their chemical structure, the CNN classification of the stain patterns was highly reproducible. Across all different polymers, the overall classification accuracy of the CNN was 96%. When challenging the CNN with images from an unknown polymer coating, i.e., poly[(4-bromo-*p*-xylylene)-*co*-(*p*-xylylene)], these surfaces were classified as halogenated or pseudohalogenated coatings with 95% accuracy. These findings confirm that the scope of surfaces that can be analyzed with this approach goes beyond polymer coatings already known to the CNN through the training procedure and validates the method as a simple, yet versatile surface analysis tool.

## Introduction

The interfacial properties of biomaterials are governing a range of important performance attributes such as cell adhesion,^1,2^ biocompatibility,^3^ and wettability.^4^ It is a common strategy to use polymer coatings to augment materials with critical surface properties such as antifouling, bactericidal, or biocompatible.^5,6^ To match the right polymer coating with a particular biomedical application, accurate surface analysis is essential for optimizing coating performance. Related, surface characterization is frequently employed to screen for potential surface contaminants or variations in surface chemistry that may affect adhesion, wetting, biological integration, surface fouling, or optical performance characteristics.^7^

**Figure 1:**
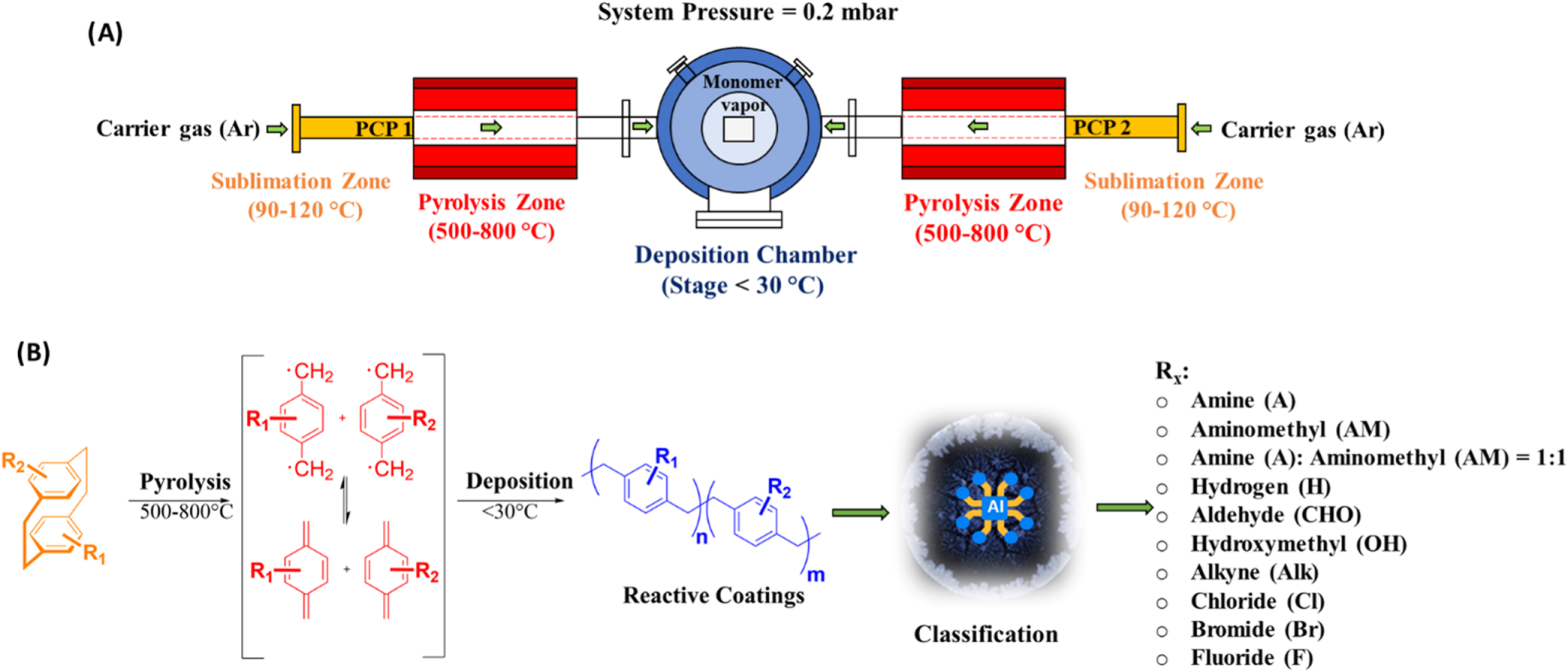
Concept and schematic depiction of CVD polymerization steps. (A) Cartoon of the CVD setup. (B) Chemical equation of the CVD polymerization of ten different PCPs used in this study. All precursors were asymmetrically functionalized (R_2_=H), except PPX-Chloride (R_1_=R_2_=Cl), PPX-Bromide (R_1_=R_2_=Br) and PPX-Fluoride (both rings fully functionalized with four F substituents each). The objective of this study is to identify a wide range of functionalized CVD coatings using a deep-learning approach.

Chemical vapor deposition (CVD) polymerization is a widely utilized technique to engineer surface properties, recognized for its effectiveness in achieving high-performance surface functionalization.^8−10^ These polymer coatings have been applied to a variety of substrates, including glass, metal, ceramic, and synthetic materials.^11^ CVD polymerization employs [2.2] paracyclophane (PCP) precursor molecules that are activated and polymerized in a three-step sequence under vacuum conditions (0.1–0.3 mbar). The initial step involves sublimating PCP at temperatures between 100–200 °C, followed by homolytic cleavage at 500–800 °C, which generates reactive 1,4-quinodimethane species.^12^ These species deposit onto a substrate at temperatures below 30 °C, where they polymerize into uniform polymer films (Figure 1A).^13^ Because CVD polymerization involves direct deposition of the polymer film from the gas phase, no solvents, catalysts, or liquid phases are needed.^14^ This results in uniform and pinhole-free coatings with minimal impurities^15^ that can easily be patterned.^16^ The stability and biocompatibility of PPX coatings are advantageous for medical devices,^17^ tissue engineering scaffolds,^18^ and drug delivery systems, ensuring minimal adverse responses while maintaining functional efficacy.^19^

CVD polymerization of functionalized PCPs can introduce a wide range of functional groups while keeping the backbone chemistry constant.^13,20^ Although the precise characterization of functionalized PPX coatings can be challenging, a wide range of surface analysis techniques^21^ have been used to characterize CVD coatings including IR spectroscopy,^22^ XPS,^23^ SIMS,^21,24,25^ contact angle,^26^ ellipsometry,^27^ AFM,^28^ and SEM.^29^ However, all these methods have some level of inherent limitations. For instance, IR measurements may lack sensitivity when analyzing samples with low thickness or functional groups associated with weak absorption bands.^30^ Overlapping absorption bands can further complicate the interpretation of IR spectra. XPS requires complex instrumentation, which is costly to acquire and maintain and often requires the help of skilled operators. Several of the more powerful analytical methods, such as XPS or TOF-SIMS, require ultrahigh vacuum heightening the overall operational complexity and cost, and limiting the scope of samples that can be analyzed. Therefore, new surface analytical methods that are straightforward to use under ambient conditions, fast, cost-effective, and accurate are needed. The deep learning algorithm bridges the gap between low-tech and traditionally established high-tech methods by offering an experimentally simple analysis protocol akin to contact angle measurements, yet with the added benefit of detailed information about the presence of functional moieties on a surface. Machine-learning approaches are emerging as powerful tools to enhance traditional surface analysis and classification methods.^31^ If large data sets are available, machine-learning algorithms can identify complex patterns and correlations that accelerate conventional analysis. Major advances are related to the introduction of pretrained CNNs, which allow for faster data processing, improved predictive accuracy, and more precise control over measurements and analysis.^32,33^

In our previous studies, deep learning methods were developed to predict single amino acid mismatches in amyloid β peptides.^34^ By analyzing stain patterns from drying droplets using deep-learning neural networks trained on polarized light microscopy (PLM) images of peptides, critical insights were gained into both primary and secondary protein structures.^34^ Furthermore, the application of the pretrained InceptionV3 network on PLM images of various DNA as well as various histone-DNA mixtures enabled DNA categorization, the assignment of various histone-DNA binding affinities,^35^ and interaction patterns of different immunoglobulins with protein A.^36^

A nonuniform deposit typically forms on the substrate as a sessile droplet of a volatile solvent containing nonvolatile solutes or particles evaporates.^37,38^ These stain patterns are characteristic, reflecting the physicochemical properties of both, the liquid and the supporting surface.^39^ This phenomenon, often referred to as the ″coffee-ring effect″, is influenced by various factors, including environmental conditions^40^ (temperature, relative humidity), the chemical composition and concentration of solute components,^41^ and the chemistry of the substrate.^42^ Thus, by careful control of these parameters, critical insights into the state of the solution or the surface can be obtained.

In this study, we seek to leverage convolutional neural network algorithms to analyze a wide variety of PPX-functionalized surfaces, which were prepared via CVD polymerization. In an automated workflow, defined volumes of Bovine Serum Albumin (BSA) solution are deposited in a massively parallel manner, followed by rapidly capturing images of the resulting stain patterns using an automated PLM (Figure 1B). Using this approach, we can produce about 200 images per hour. Finally, we demonstrate the utility of a pretrained InceptionV3 model to analyze these large sets of images obtained from drying BSA solution on various functionalized surfaces.

## Results and Discussion

To produce uniform and functionalized substrates, we employed CVD polymerization of PPX (Figure 1).^43^ Glass wafers were coated with ten different PPX films, incorporating a range of functional groups (Figure 1). To ensure data comparability and interpretability, we standardized the microscope settings by maintaining consistent resolution, objective magnification (10x), and exposure time throughout the process. Environmental conditions, including humidity and temperature, were carefully controlled, and homogeneous surfaces were used. Additionally, droplets were randomly placed on different batch-coated plates to enhance the reliability of the results. The coating thicknesses varied between 50–60 nm. A constant volume of 2 μL of an aqueous BSA solution in buffer was applied to each coated surface and allowed to dry under controlled conditions (40% humidity, 23 °C) for 45 ± 5 min. As the BSA solution dries and reaches saturation, the protein components precipitate, starting at the droplet’s edge and moving toward the center, resulting in at least partially crystalline stains. Coupled evaporation and crystallization processes in protein droplets lead to pattern formation that still initiates from the contact line, but gradually progresses toward the center of the droplet. TOF-SIMS analysis confirms that the protein as well as the salt (buffer) are deposited throughout the entire stain pattern (Supporting Figure S1).^34−36^ We rely on PLM to generate the CNN input data to adequately capture the crystalline salt structures, with the protein playing an important role in facilitating salt crystal morphology. The Grad-CAM visualizations in Supporting Figure S2 confirm that both the edge and central regions are recognized by the CNN.

**Figure 2:**
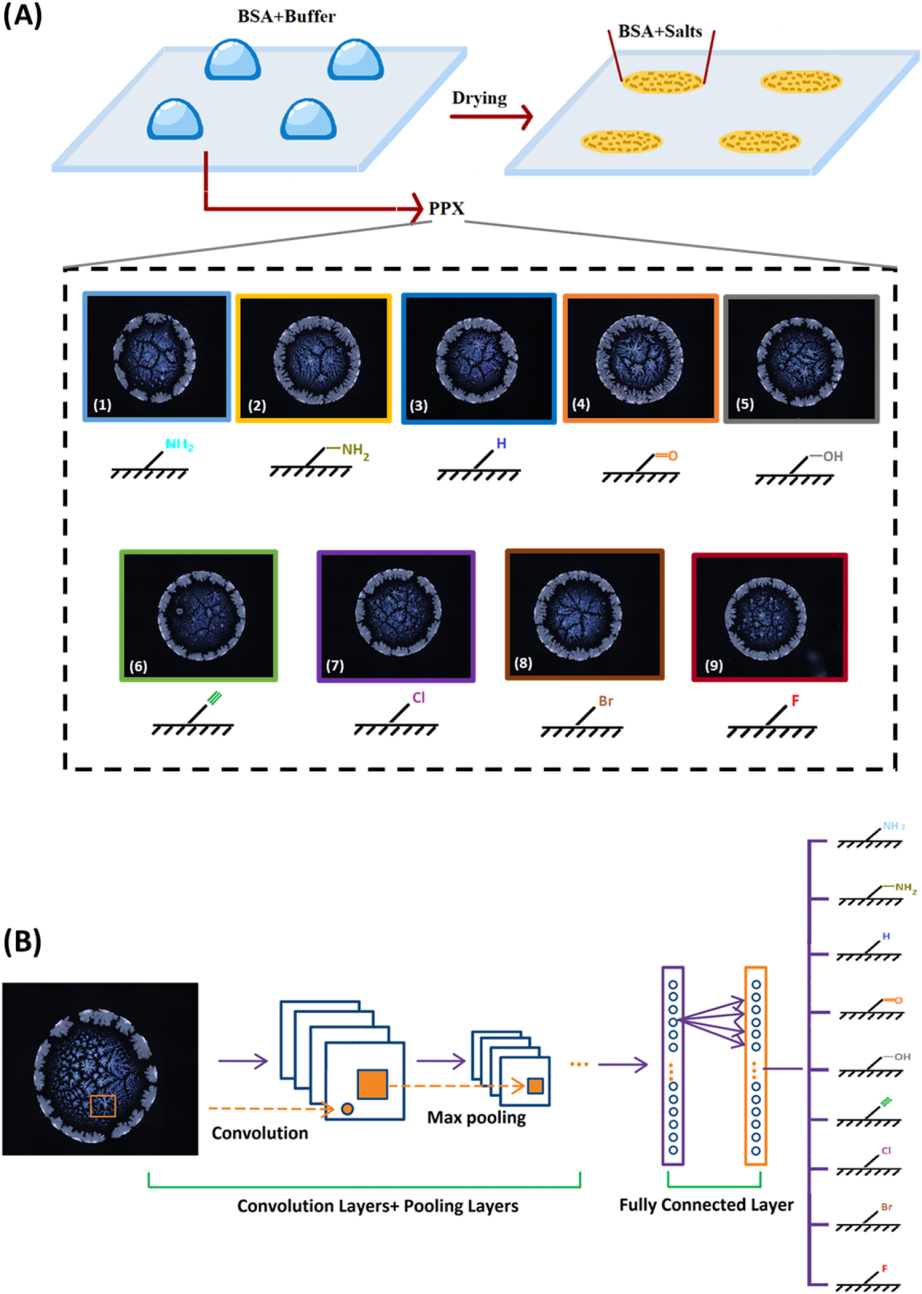
Classification of surface polymer chemistries by image analysis of deposition droplets using a deep learning (DL) method. (A) Representative PLM images of deposition patterns of BSA on nine PPX-coated glass wafers. Stains were obtained by depositing 2 μL droplets of 0.1 mg/mL BSA dissolved in an aqueous carbonate-bicarbonate buffer with sodium chloride (pH = 9.2) solution. (B) The pretrained InceptionV3 network was applied with a 5063 number of PLM images of BSA stains dried on various polymer surfaces for image classification. The trained network was able to classify various polymer surface chemistries as well as predict the similarity between unknown testing images and those in the training data set.

Figure 2A displays PLM images depicting a typical drying pattern obtained from BSA solution dried on nine different PPX-coated surfaces including PPX-Amine (A), PPX-Aminomethyl (AM), PPX-Hydrogen (H), PPX-Aldehyde (CHO), PPX-Hydroxymethyl (OH), PPX-Alkyne (Alk), PPX-Chloride (Cl), PPX-Bromide (Br), and PPX-Fluoride (F). The image of each dried droplet was captured with dimensions of 2344 × 1878 pixels in JPG format. As is apparent from Figure 2A, the BSA patterns on each surface were undistinguishable to the naked eye. To ensure an unbiased classification of polymer surfaces, we pretrained the InceptionV3 network with 400–500 images per group. InceptionV3 was applied due to its high accuracy and faster training time compared to other advanced CNNs like NasNetLarge.^34,44^Figure 2B illustrates the architecture of the InceptionV3 network. An average of 480 images per surface group were used for the training and validation set (comprising about 85% of the total images), while 80 images (about 15% of the total images) were reserved for the test set (which were not exposed to the trained network).

Upon completion of the training, the network was used to achieve two objectives: (i) classification of various surface polymer chemistries based on the analysis of dried BSA patterns, and (ii) predict and classify unknown surfaces using the trained model.

**Figure 3:**
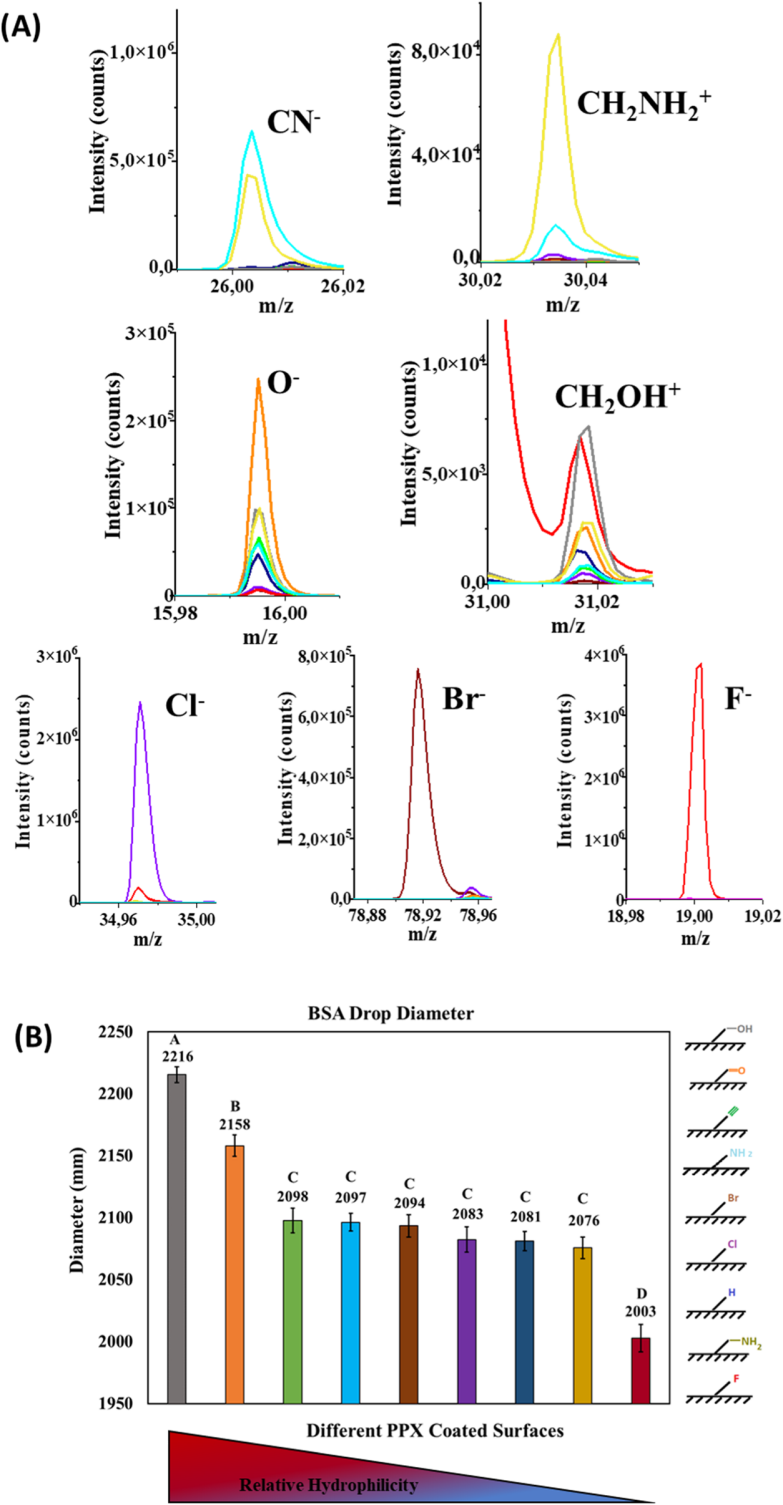
Surface characterization of 9 functionalized polymer coatings prepared via CVD polymerization. (A) TOF-SIMS analysis. (B) Stain diameters of various CVD coatings based on image analysis (mean value, *N* = 150, error bars represent the standard error). Groups sharing the same letters indicate no statistically significant differences according to Tukey’s test.

Initially, we rigorously characterized the nine polymer coatings using a combination of TOF-SIMS, FTIR, and contact angle measurements. Figure 3A displays the TOF-SIMS analysis of PPX-coatings with varying functionalization. Except PPX-Alk, all functional groups have characteristic heteroatoms and could be identified by this technique. The halogenated polymer films of PPX-F, PPX-Cl and PPX-Br were each uniquely characterized by the presence of characteristic F^–^, Cl^–^ and Br^–^ fragments, respectively. The presence of oxygen-containing fragments identified PPX-CHO and PPX–OH coatings, whereby the most intense O^–^ signal was found for PPX-CHO, while the most intense CH_2_OH^+^ signal was found for PPX–OH. We note that low-level oxygen signals can be ambivalent as they can also be detected for other PPX films due to the postpolymerization quenching of free radicals with molecular oxygen. Both PPX-AM and PPX-A were identified by nitrogen-containing fragments that presented as CN^+^ in similar intensity, but the intensity of the CH_2_NH_2_^+^ signal was higher for PPX-AM due to the additional methylene group in this polymer. The entire spectra of the spectral range between 10 and 85 *m*/*z* can be found in the Supporting Information (Figure S3).

The relative hydrophobicity (wettability) of various PPX surfaces was assessed by measuring the size of dried droplets (average of 150 measurements) on each PPX-coated surface (Figure 3B). Based on the statistical analysis, the hydrophobicity of the compounds was ranked in the following order: PPX–OH < PPX-CHO < PPX-Alk ∼ PPX-A ∼ PPX-Br ∼ PPX-Cl ∼ PPX-H ∼ PPX- AM< PPX-F.

Infrared Reflection Absorption Spectroscopy (IRRAS) was employed to confirm the presence and nature of the respective functional groups associated with each coated surface (Figure S4).

### Surface Classification and Recognition

**Figure 4:**
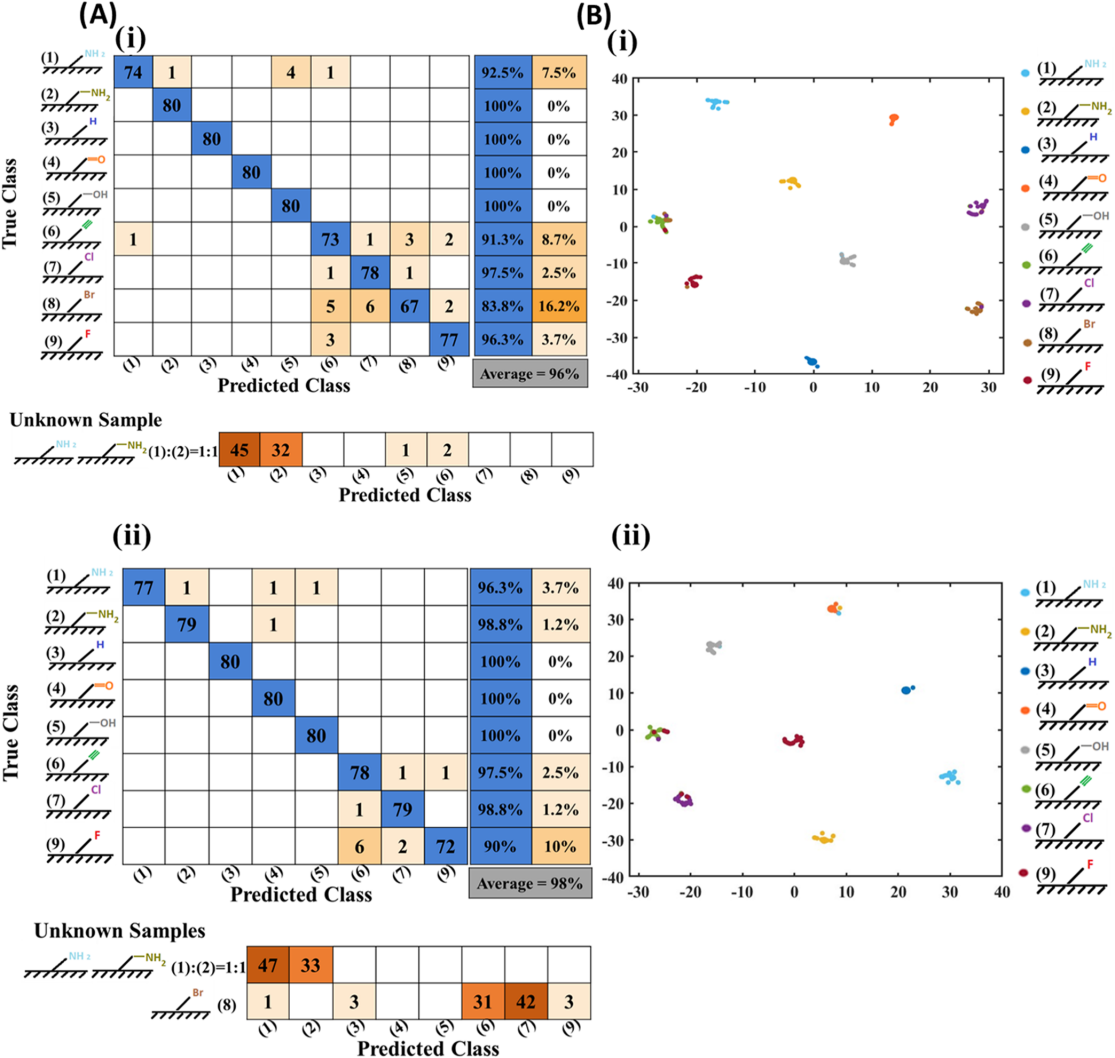
CNN classification of functionalized CVD coatings based on images of their respective stain patterns. (A) DL-informed surface recognition of PPX-coated glass wafers where pretraining was done (i) with and (ii) without the inclusion of images from PPX-Br. Deposition stains of BSA were obtained by depositing 2 μL droplets of an aqueous 100 mM carbonate-bicarbonate buffer (pH 9.2) with a 50 mM sodium chloride solution. The parameters were fine-tuned across all layers with a global learning rate of 0.001, a minimum batch size of 32 images, and up to 60 epochs. The pretrained InceptionV3 networks, with and without PPX-Br, achieved validation accuracies of 95% and 96%, respectively. (B) t-SNE plot illustrating the outcomes from the ″SoftMax Activation″ layer of the trained CNN. The t-SNE analysis was conducted using a MATLAB machine-learning package with a perplexity of 30 and a learning rate of 500.

A total of 5,063 PLM images of BSA stains dried on various polymer surfaces were prepared for classification using the pretrained InceptionV3 network. The analysis included all images without any form of selection. In general, the CNN demonstrated an average training accuracy of 96% for the nine different PPX-coated surfaces, suggesting that the pretrained network could distinguish polymer surfaces with high accuracy. As depicted in Figure 4, the CNN results revealed that PPX–OH was identified with 100% prediction accuracy. It was also the polymer coating with the highest relative hydrophilicity, a factor that has previously been shown to influence BSA surface adsorption.^45^ Similarly, the PPX-CHO coated surface was classified with 100% accuracy (Figure 3B). The observed results are in line with past observations, such as the recent work by Sarkar et al.,^45^ that observed multilayer vs monolayer absorption of albumin on substrates with various hydrophobicity. In our case, even minuscule differences in the surface chemistry give raise to unique protein stain patterns, which can be recognized and categorized by the CNN.

As mentioned above, PPX-Alk, PPX-A, PPX-Br, PPX-Cl, PPX-H, and PPX-AM exhibited similar relative hydrophobic properties (Figure 3B). The highest number of misclassifications occurred between halogenated surface coatings, with the lowest prediction accuracy (83.8%) associated with PPX-Br. The majority of misclassifications (13.7%) of the PPX-Br group were identified by the CNN as PPX-Cl and PPX-Alk groups. Both are halogenated or pseudohalogenated coatings that exhibited similar hydrophobicity to PPX-Br. Although PPX-F exhibited higher hydrophobicity compared to other functionalized coatings, the CNN confused PPX-F coatings for other halogenated PPX coatings as well as PPX-Alk. This influence on the network’s decision was detected in the Grad-CAM analysis (Figure S2). Background interference and contrast from stain-free areas were the main contributors to these misclassifications.

The second lowest prediction accuracy was observed for PPX-Alk (91.3%): approximately 7.5% of the images were misclassified as halogenated PPX surfaces. The CNN results suggest that the predominant misclassifications between alkyne and halogen groups are consistent with their similar hydrophobic properties. The prediction accuracy for PPX-A was 92.5%, with 2.5% misclassification relative to groups with similar hydrophobicity, and 5% misclassification as PPX–OH coatings (Figure S2).

Next, we assess the model’s ability to predict unknown PPX-A/AM copolymer surfaces, i.e., novel polymer coatings that were not included in the training set. The pretrained network accurately predicted the unknown samples predominantly with amine (56%) and aminomethyl groups (40%) (Figure 4Ai). Moreover, we evaluated our trained network using a small number of unknown PPX-Br samples collected from coated surfaces that were not included in the training set. The results demonstrated high prediction accuracy for the “underpowered” study, which were in line with the original study (Figure S5).

In the following, as depicted in Figure 4Aii, PPX-Br was omitted from the training set, and the network was only trained on the remaining eight PPX-coated surfaces. As expected, the average prediction accuracy across all eight groups increased. Furthermore, a similar trend of misclassification (Figure 4Ai) was observed between PPX-Alk and halogenated PPXs, which showed the highest number of misclassifications among all groups. We then added images from the unknown PPX-A/AM copolymer and PPX-Br to the test data set. The results indicate that 100% of the unknown PPX-A/AM binary copolymer samples were classified as either PPX-A or PPX-AM. For the unknown PPX-Br coated surfaces, 56% of the images were categorized as halogenated PPX, while 96% of the total images were classified as polymer films, which had similar hydrophobicity to PPX-Br (Figure 4Aii). The t-SNE algorithm revealed distinct clusters in BSA stain images from nine different polymer surfaces, confirming that the stain patterns were highly reproducible and unique to each surface type (Figure 4Bi,4Bii). The t-SNE results with perplexities of 10, 50, and 100 consistently show clear clustering, indicating that the network has learned robust, discriminative features and captures meaningful relationships at both local and global levels (Figure S6). The Grad-CAM results indicated that the CNN was mostly trained using patterns in the center of the stains, rather than the edge patterns, suggesting that the stain size had a lower influence on surface recognition (Figure S7). During training, the model achieved 100% accuracy on the training set, with slightly lower, yet stable validation accuracy, indicating strong generalization. The alignment of training and validation loss trends further confirmed the absence of overfitting. Additionally, the test accuracy remained comparable to the validation accuracy across all cases, reinforcing the model’s ability to generalize to new data. The training and validation accuracies over the training epochs are presented in the new Figure S8. Precision, recall, and F1 scores are presented in Tables S1 and S2.

### Effect of Ionic Strength on CNN Classification

**Figure 5:**
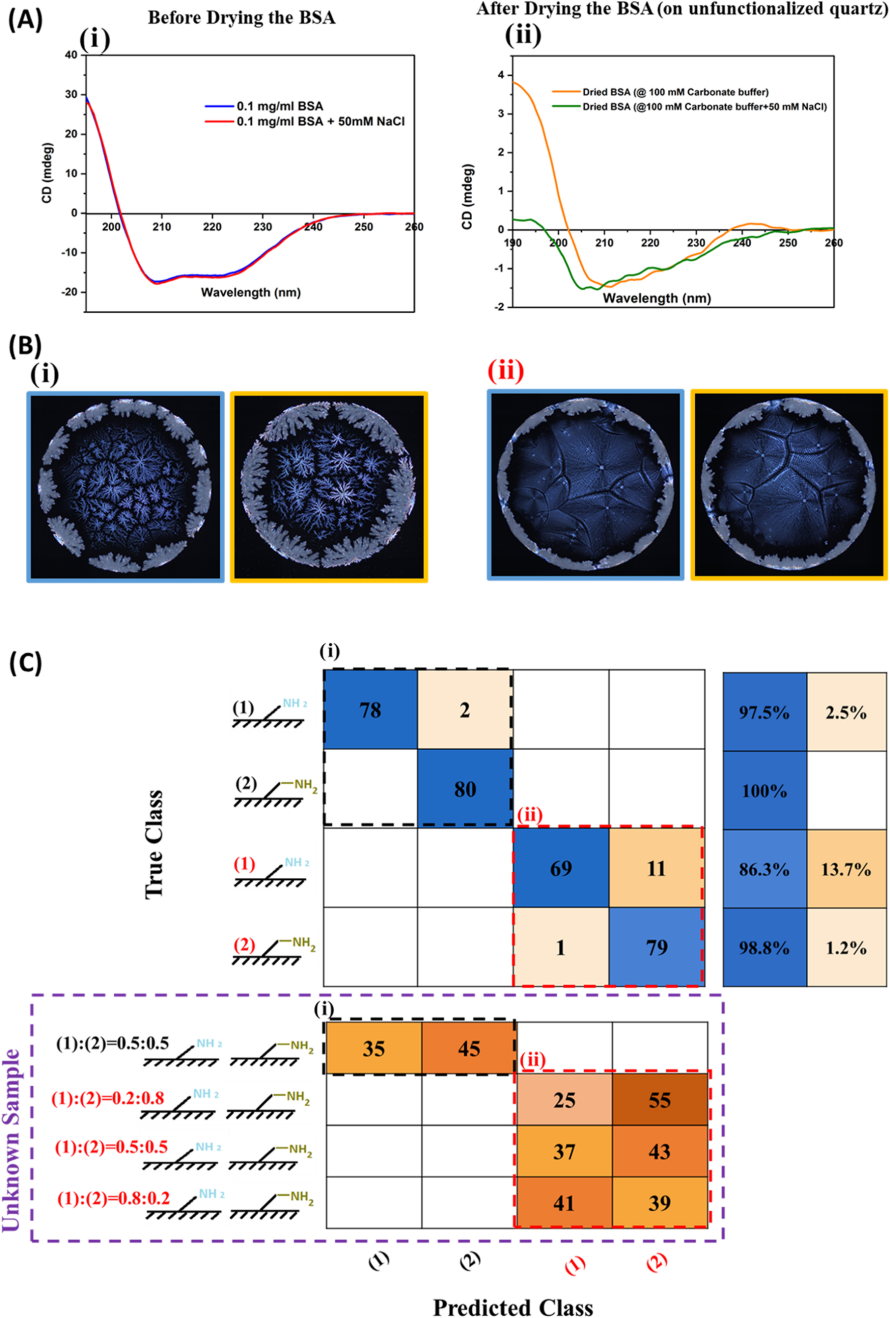
Effect of ionic strength on protein secondary structure and the relevance for classification of PLM images of deposition patterns of BSA on amine and aminomethyl. (A) Effect of ionic strength on secondary structure changes of (i) BSA dissolved in diluted carbonate-bicarbonate buffer with and without sodium chloride, and (ii) Effect of drying process on BSA secondary structure (on quartz substrate in absent and presence of sodium chloride), studied by CD spectroscopy. (B) Stains were obtained by depositing 2 μL droplets of an aqueous (i) 100 mM carbonate-bicarbonate buffer (pH 9.2) with 50 mM sodium chloride solution, and (ii) 100 mM carbonate-bicarbonate buffer (pH 9.2) solution. (C) DL-informed effect of ionic strength on surface recognition. The confusion chart was obtained for stains of BSA droplets dissolved in an aqueous carbonate-bicarbonate buffer (pH 9.2), (i) with 50 mM sodium chloride and (ii) without sodium chloride solution onto amine and aminomethyl coated glass wafers. The parameters were fine-tuned across all layers with a global learning rate of 0.001, a minimum batch size of 32 images, and up to 60 epochs. The pretrained InceptionV3 network achieved test and validation accuracies of 96% and 95%, respectively.

To investigate the impact of ionic strength on the surface classification by the CNN, the surfaces of PPX-A and PPX-AM were compared in the presence and absence of sodium chloride. Sarkar et al. showed that the ionic strength significantly influences BSA adsorption. In the absence of ions, a tilted monolayer of globular BSA molecules forms on both hydrophilic and hydrophobic surfaces, with greater tilting on hydrophobic surfaces, leading to a denser layer.^45^ It is important to note that the secondary structure of BSA, dissolved in a diluted carbonate-bicarbonate buffer, was unaffected by an ionic strength of 50 mM as examined by CD spectroscopy. However, when BSA was dried on a quartz crystal surface, 50 mM sodium chloride resulted in a higher prevalence of random coil structures in the CD spectrum of BSA (Figure 5A).

Next, the sodium chloride was removed from the final buffer solution, which was composed of only 100 mM carbonate-bicarbonate buffer (pH 9.2). The emerged patterns with and without the addition of sodium chloride were clearly distinguishable (Figure 5Bi,5Bii). For each group (amine and aminomethyl), approximately 80% of the total images were allocated to the training and validation sets, while the remaining 20% were used for the testing set. Figure 5C displays the confusion matrices for each category with (i) and without (ii) the addition of sodium chloride. The results indicate that with decreasing ionic strength, images from each category became less distinguishable, and the overall prediction accuracy decreased from 99% (with NaCl) to 93% (without NaCl). Moreover, we evaluated the network’s performance using unknown samples (BSA dissolved in carbonate buffer (i) with and (ii) without additional NaCl, deposited onto substrates coated with PPX A/MA ratios of 0.2/0.8, 0.5/0.5, and 0.8/0.2). For unknown samples on PPX A/MA = 0.5/0.5, 56% of the images were classified as PPX-MA surface, and 44% were classified as PPX-A surface. Additionally, the trained network successfully distinguished these samples from others with lower ionic strength, achieving 100% accuracy. Similarly, for unknown samples in the absence of additional NaCl, we evaluated the network’s performance with three copolymer surfaces at different PPX A/MA ratios: 0.2/0.8, 0.5/0.5, and 0.8/0.2. For the (0.5/0.5) copolymer, 54% of the samples were classified as PPX-MA, and 46% as PPX-A. For other ratios (PPX A/MA = 0.2/0.8 and 0.8/0.2), the majority of the samples were classified into the corresponding groups: 69% of PPX A/MA = 0.2/0.8 samples were classified as PPX-MA (since 80% of the copolymer consists of PPX-MA), while 31% were classified as PPX-A (since 20% of the sample consists of PPX-A). The training and validation accuracies over the training epochs is presented in Figure S9. The precision, recall, and F1 score were presented in Table S3.

The relative hydrophobicity of each functionalized surface was evaluated in the absence and presence of 50 mM sodium chloride, 150 images of each surface were randomly collected (Figure S10).

### Effect of Image Rotation on Surface Classification

The consideration of bias is an important aspect when interpreting deep-learning based analysis methods.^46^ In particular, we were concerned that the direction of observation could influence the outcomes of the CNN analysis. To assess the presence of geometric training bias, the test set images were rotated at two different angles and directions, and the classification accuracy of surface chemistries was evaluated using the pretrained network.

**Figure 6:**
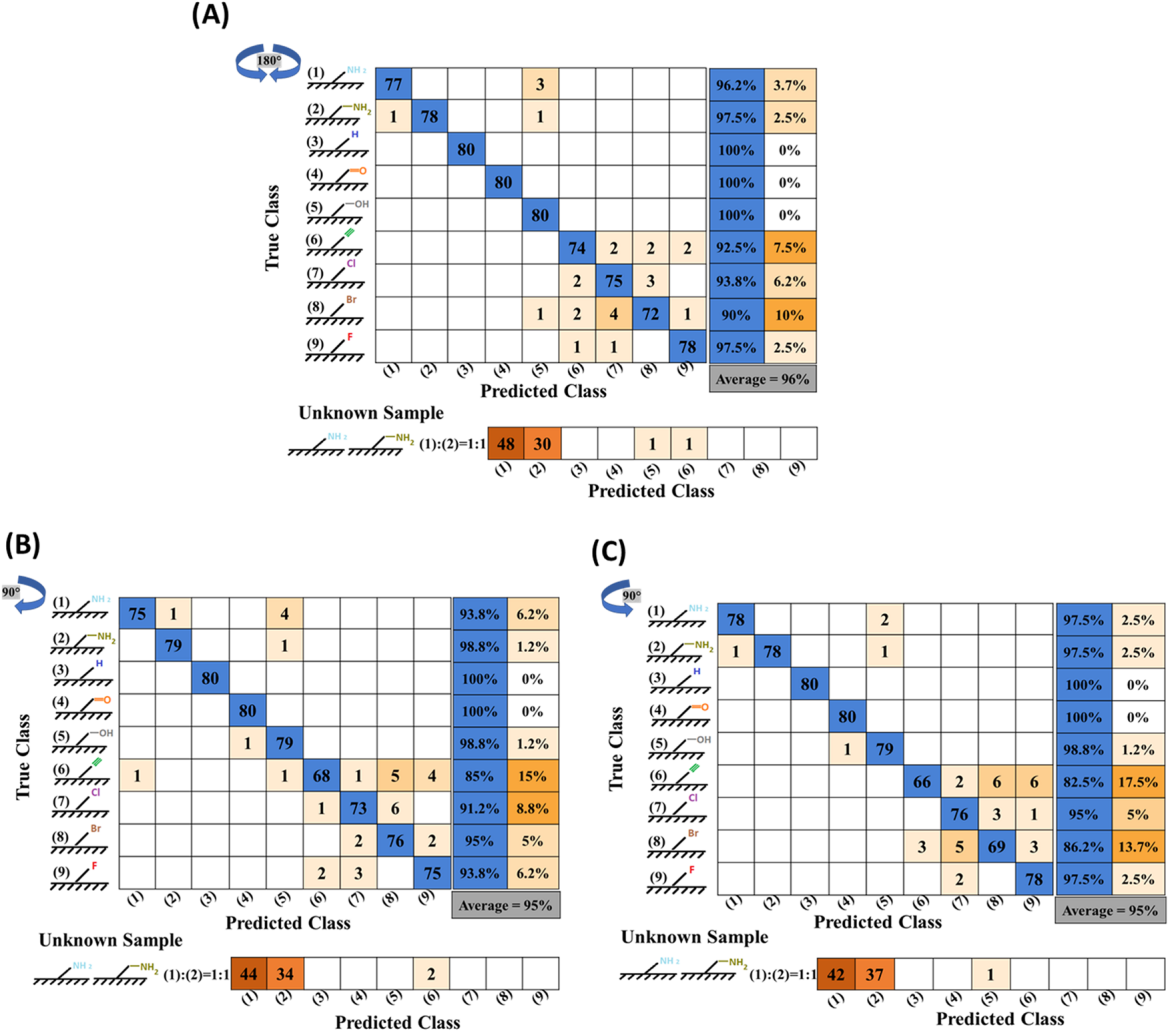
Evaluation of geometric bias in the CNN analysis: (A) 180-degree rotation clockwise and counterclockwise, (B) 90-degree rotation clockwise, and (C) 90-degree rotation counterclockwise.

The trained model (with the original images) was tested after systematically rotating the images from each surface category. The test images were rotated by (A) 180 deg and (B,C) 90 deg in both clockwise and counterclockwise directions (Figure 6). The 180-degree rotation in both directions produced identical confusion charts, with no change in average prediction accuracy compared to the unrotated case (96%). In the case of 90-degree rotation, both clockwise and counterclockwise, the average prediction accuracy decreased by 1% (to 95%), which is negligible. Additionally, in all three rotation scenarios, the misclassification trend remained constant compared to the unrotated case.

After a 180-degree rotation in both directions, the unknown sample had a prediction accuracy of 97.5%. For 90-degree rotations, the prediction accuracies were 97.5% in the clockwise direction and 98.75% in the counterclockwise direction. In general, the results showed that the classification accuracy was independent of the angle of rotation (90 and 180 deg), indicating the network’s ability to generalize and accurately classify surfaces regardless of image orientation.

## Conclusions

This study utilizes deep learning techniques to identify the chemical functionalization of surfaces with various CVD coatings. By analyzing BSA stain images with the InceptionV3 CNN, highly accurate and predictive classification and identification of functional groups was achieved. Higher prediction accuracy was obtained in the presence of additional ionic strength, which corroborates with the slight destabilizing effect of ionic strength on the BSA secondary structure. Moreover, the results suggest that image rotation does not affect prediction accuracy, excluding the potential for geometric bias in the network training. The approach introduced in this study could enable the swift, cost-effective, and relatively straightforward evaluation of surface chemistry candidates, offering significant potential for widespread use in surface engineering. These findings suggest that the foundational methodology is not limited to CVD-based polymer surfaces. This work has the potential to be applied to functionalized polymer surfaces more broadly, provided a sufficiently diverse and representative data set is available for training. As this approach is extended to a wider range of polymer coatings, requirements to be considered during the selection process include low roughness, homogeneous coating, transparency, and stability against water. Since the performance of materials in various environments and applications is closely linked to their surface characteristics, protein-material interactions could be studied using this approach. Identifying functional groups as distinctive markers on a material’s surface allows researchers to forecast and tailor surface properties for specific biomedical applications.^47^ This study underscores the flexibility and efficacy of transfer learning and fine-tuning, showcasing robust performance in challenging scenarios, including the differentiation of chemically similar samples. Future investigations could focus on exploring alternative architectures to further validate and extend the generalizability of these promising results.

## Material and Methods

### Bovine Serum Albumin (BSA) Solutions

BSA was prepared from VWR. To prepare the BSA solution, the lyophilized protein was dissolved in a 100 mM carbonate-bicarbonate buffer to a concentration of 0.1 mg/mL. This buffer, with a pH of 9.2, was made using ultrapure water from a Milli-Q Plus system (Millipore, Schwalbach, Germany) and contained 91 mM NaHCO_3_ and 9 mM Na_2_CO_3_ (Merck Chemicals GmbH). The protein solution was mixed for 30 min at room temperature using an SB3 tube rotator (Stuart, Stone, UK) set at 10 rpm and was then stored in aliquots at −20 °C.

### Surface Preparation through Chemical Vapor Deposition Polymerization

The glass wafers, sized to 120 mm × 80 mm and with a thickness of 0.1 ± 0.05 mm (Optrovision, München, Germany), were subjected to cleansing using a plasma cleaner (Tergeo, Union City, CA). In each deposition batch, two slides of glass substrate were put in the deposition chamber. Adjacent to it, two silicon (Si) wafers were placed to gather data about the thickness of the coating. The deposition speed was determined and could be adjusted to a rate of 0.3–0.5 Å/s by altering the sublimation temperature of the precursor, using a quartz crystal detector situated within the deposition chamber. Functionalized PCP was obtained from commercial sources (PCP-H (Curtiss-Wright Surface Technologies, Galway, Ireland), PCP-Cl (SCS GmbH, Surrey, UK), PCP-F (TCI Deutschland GmbH, Eschborn, Germany)) or synthesized in-house via previously published protocols.^48^ These precursors were sublimed under vacuum and then converted by pyrolysis into quinodimethane, which polymerized spontaneously upon condensing onto the glass surface. An argon flow of 20 sccm was maintained as the carrier gas. The sublimation temperature ranged from 100–110 °C, followed by pyrolysis at 660 °C for PCP-H, PCP-Cl, PCP-Br, PCP-F, PCP-CHO, PCP-A, PCP-AM, and PCP-Alk. For PCP–OH, the pyrolysis temperature was set at 540 °C. The coating pressure was maintained at 0.2 mbar. The copolymer synthesis was carried out using a two-source setup.

### Droplet Deposition

An automated 96-well microplate pipetting system (epMotion 5070, Eppendorf AG, Hamburg, Germany) with a 1-channel dispenser (TS10, Eppendorf AG, Hamburg, Germany) was utilized for precise deposition of droplets onto glass slides. The system was housed in a climate chamber (ICH 750, Memmert GmbH + Co. KG, Schwabach, Germany), maintaining conditions at 23 °C ± 0.5 °C and 45% ± 3% humidity. Each droplet, with a volume of 2 μL, was dispensed at a rate of 3 mm/s, with the system programmed to place 96 droplets in an 8-row by 12-column array per glass plate. After drying for 45 ± 5 min, deposition patterns were imaged using a polarized light microscope (Olympus BX-53F, Tokyo, Japan) with an automated stage. Images were captured at a uniform light intensity using a 10× objective, then stitched together with the multi-image alignment (MIA) algorithm in CellSens software (Olympus, Tokyo, Japan), resulting in JPG format images sized 2344 × 1878 pixels.

### Convolution Neural Network Training and Testing Set

The training and analysis of PLM images were conducted using MATLAB software (R2022a, MathWorks Inc.), with Inception V3, a pretrained CNN network, chosen for its quick performance and reliable accuracy. Developed by Google, Inception V3 is a deep convolutional neural network designed for image classification, featuring ″Inception modules″ to capture multiscale image features efficiently and consisting of 315 layers. Input images were resized to 299 × 299 pixels for preprocessing before being used for training and inference. Using a transfer learning approach, the network was fine-tuned with a smaller set of new images, while the final classification layer was retrained with this new data set. Fine-tuning involved adjusting all layers with a global learning rate of 0.001 and a minimum batch size of 32 images. To prevent overfitting and ensure the network does not memorize the training data, images were augmented with random horizontal and vertical reflections, each with a 50% probability, for all trained networks. Accuracy serves as a key metrics for evaluating the performance of classification models, and a confusion matrix offers a comprehensive overview of the algorithm’s prediction outcomes. To further evaluate the classification performance of the image-based InceptionV3 network, precision, recall, and F1 score were calculated. To demonstrate the network’s clustering capability, the t-distributed Stochastic Neighbor Embedding (t-SNE) algorithm was applied to the “Softmax” layer of the trained CNN. Additionally, the gradient-weighted class activation mapping (Grad-CAM) algorithm was used to identify and visualize the image regions that most influenced the network’s classification decisions.

### Mass Spectrometry

ToF-SIMS was performed on a TOF.SIMS5 instrument (ION-TOF GmbH, Münster, Germany). This spectrometer is equipped with a field emission bismuth cluster primary ion source and a reflectron type time-of-flight analyzer. The main chamber pressure was 5 × 10^–9^ mbar. For high mass resolution, the Bi source was operated in the “high current bunched” mode providing short Bi_3_^+^ primary ion pulses at 25 keV energy and a lateral resolution of approximately 5 μm. The short pulse length of 1.1 ns allowed for high mass resolution. Primary ion doses were kept below 2 × 10^11^ ions cm^–2^ (static SIMS limit) for all measurements. Spectra were calibrated on the omnipresent CH^–^, C_2_^–^, C_2_H^–^, OH^–^; or on the CH^+^, CH_2_^+^, CH_3_^+^, and C_2_H_3_^+^ peaks. Spectra were normalized by the total ion dose.

### Infrared Reflection–Absorption Spectroscopy (IRRAS)

Infrared spectral analysis of the polymer films was conducted using a Bruker VERTEX 80 FTIR (Bruker Optik GmbH, Ettlingen, Germany). The device features a horizontal reflection unit for measurements in grazing incidence reflection mode with an 80° incident angle to the surface normal. The spectra were scanned with a resolution of 2 cm^–1^ across the range of 500 to 4000 cm^–1^. Background correction was carried out using the onboard Bruker OPUS software.

### CD Spectroscopy

The far-UV CD spectra of the peptide solutions were captured using a J-1500 spectropolarimeter (JASCO, Germany) at a temperature of 20 °C. For the solution samples it was conducted in quartz glass cuvettes with a 1 mm optical path length (Suprasil, Hellma Optik GmbH, Jena, Germany) within the wavelength range of 260 to 190 nm, with measurements taken at 0.5 nm intervals. Each sample underwent two repeated scans at a scan rate of 100 nm min^–1^, an 8 s response time, and 8 nm bandwidth. The obtained data were averaged for each sample, along with its respective baseline obtained from the protein-free sample. For protein samples on solid surface, the quartz glass (Chemglass life science) was used. The protein concentration used was 0.1 mg mL^–1^ in a 20 mM carbonate-bicarbonate buffer, with and without 50 mM at pH 9.2.

### Statistical Analysis

The analysis of variance was performed to measure relative surface hydrophobicity using the Tukey method with Origin software (2022b). The Tukey method was applied to identify significant differences, with the significance level set at *p* < 0.05.

## Supporting Information Available

ToF-SIMS Survey spectra of negative and positive polarity measurements; IRRAS of various PPXs; Grad-CAM activation maps illustrating misclassified PLM images; Classification performance of a pretrained network on unseen PPX-Br patterns; t-SNE visualizations of the feature space with perplexity values of 10, 50, and 100; Grad-CAM activation maps, accuracy and loss curves for training and validation per epoch in surface recognition of PPX-coated glass wafers; accuracy and loss curves per epoch for training and validation in network training on the effect of ionic strength in surface recognition; wettability of PPX-A and PPX-AM functional surfaces; precision, recall, and F1-score derived from the confusion matrix for the network trained with 9 functionalized CVD coating surfaces; precision, recall, and F1-score derived from the confusion matrix for the network trained with 8 functionalized CVD coating surfaces (excluding PPX-Br); precision, recall, and F1-score derived from the confusion matrix for the network trained to evaluate the effect of ionic strength on functionalized surface classification (PDF)

## References

[ref1] Chang T. Y.; Yadav V. G.; De Leo S.; Mohedas A.; Rajalingam B.; Chen C.-L.; Selvarasah S.; Dokmeci M. R.; Khademhosseini A. Cell and protein compatibility of parylene-C surfaces. Langmuir 2007, 23 (23), 11718–11725. 10.1021/la7017049. 17915896

[ref2] Harnett E. M.; Alderman J.; Wood T. The surface energy of various biomaterials coated with adhesion molecules used in cell culture. Colloids Surf., B 2007, 55 (1), 90–97. 10.1016/j.colsurfb.2006.11.021. 17207976

[ref3] Golda-Cepa M.; Kulig W.; Cwiklik L.; Kotarba A. Molecular Dynamics Insights into Water–Parylene C Interface: Relevance of Oxygen Plasma Treatment for Biocompatibility. ACS Appl. Mater. Interfaces 2017, 9 (19), 16685–16693. 10.1021/acsami.7b03265. 28459527

[ref4] Li X.-M.; Reinhoudt D.; Crego-Calama M. What do we need for a superhydrophobic surface? A review on the recent progress in the preparation of superhydrophobic surfaces. Chem. Soc. Rev. 2007, 36 (8), 1350–1368. 10.1039/b602486f. 17619692

[ref5] Sun W.; Liu W.; Wu Z.; Chen H. Chemical surface modification of polymeric biomaterials for biomedical applications. Macromol. Rapid Commun. 2020, 41 (8), 1900430 10.1002/marc.201900430. 32134540

[ref6] Krishna D. N. G.; Philip J. Review on surface-characterization applications of X-ray photoelectron spectroscopy (XPS): Recent developments and challenges. Appl. Surf. Sci. Adv. 2022, 12, 100332 10.1016/j.apsadv.2022.100332.

[ref7] Mei H.; Laws T. S.; Terlier T.; Verduzco R.; Stein G. E. Characterization of polymeric surfaces and interfaces using time-of-flight secondary ion mass spectrometry. J. Polym. Sci. 2022, 60 (7), 1174–1198. 10.1002/pol.20210282.

[ref8] Chen H.-Y.; Hirtz M.; Deng X.; Laue T.; Fuchs H.; Lahann J. Substrate-independent dip-pen nanolithography based on reactive coatings. J. Am. Chem. Soc. 2010, 132 (51), 18023–18025. 10.1021/ja108679m. 21138264

[ref9] Jiang X.; Chen H. Y.; Galvan G.; Yoshida M.; Lahann J. Vapor-based initiator coatings for atom transfer radical polymerization. Adv. Funct. Mater. 2008, 18 (1), 27–35. 10.1002/adfm.200700789.

[ref10] Nandivada H.; Chen H. Y.; Lahann J. Vapor-based synthesis of poly [(4-formyl-p-xylylene)-co-(p-xylylene)] and its use for biomimetic surface modifications. Macromol. Rapid Commun. 2005, 26 (22), 1794–1799. 10.1002/marc.200500449.

[ref11] Kausar A. Polymer coating technology for high performance applications: Fundamentals and advances. J. Macromol. Sci., Part A 2018, 55 (5), 440–448. 10.1080/10601325.2018.1453266.

[ref12] Venkidasubramonian G.; Kratzer D.; Trouillet V.; Zydziak N.; Franzreb M.; Barner L.; Lahann J. Surface-initiated RAFT polymerization from vapor-based polymer coatings. Polymer 2018, 150, 26–34. 10.1016/j.polymer.2018.06.073.

[ref13] Plank M.; Berardi A.; Welle A.; Sauter E.; Krolla P.; Haret C.; Koenig M.; Stahlberger M.; Hassan Z.; Oßwald S.; et al. Photo-Arbuzov Reactions as a Broadly Applicable Surface Modification Strategy. Adv. Funct. Mater. 2024, 34, 2403408 10.1002/adfm.202403408.

[ref14] Lahann J. Reactive polymer coatings for biomimetic surface engineering. Chem. Eng. Commun. 2006, 193 (11), 1457–1468. 10.1080/00986440500511619.

[ref15] Lahann J. Vapor-based polymer coatings for potential biomedical applications. Polym. Int. 2006, 55 (12), 1361–1370. 10.1002/pi.2098.

[ref16] Suh K. Y.; Langer R.; Lahann J. A novel photoderinable reactive polymer coating and its use for microfabrication of hydrogel elements. Adv. Mater. 2004, 16, 1401–1405. 10.1002/adma.200400101.

[ref17] Kumar R.; Kratzer D.; Cheng K.; Prisby J.; Sugai J.; Giannobile W. V.; Lahann J. Carbohydrate-based polymer brushes prevent viral adsorption on electrostatically heterogeneous interfaces. Macromol. Rapid Commun. 2019, 40 (1), 1800530 10.1002/marc.201800530. 30368953

[ref18] Li X.; Wang L.; Yu X.; Feng Y.; Wang C.; Yang K.; Su D. Tantalum coating on porous Ti6Al4V scaffold using chemical vapor deposition and preliminary biological evaluation. Mater. Sci. Eng., C 2013, 33 (5), 2987–2994. 10.1016/j.msec.2013.03.027. 23623123

[ref19] Alf M. E.; Asatekin A.; Barr M. C.; Baxamusa S. H.; Chelawat H.; Ozaydin-Ince G.; Petruczok C. D.; Sreenivasan R.; Tenhaeff W. E.; Trujillo N. J.; et al. Chemical vapor deposition of conformal, functional, and responsive polymer films. Adv. Mater. 2010, 22 (18), 1993–2027. 10.1002/adma.200902765. 20544886

[ref20] Ross A. M.; Zhang D.; Deng X.; Chang S. L.; Lahann J. Chemical-vapor-deposition-based polymer substrates for spatially resolved analysis of protein binding by imaging ellipsometry. Anal. Chem. 2011, 83 (3), 874–880. 10.1021/ac102535j. 21226461 PMC3061569

[ref21] Ma Z.; Mao Z.; Gao C. Surface modification and property analysis of biomedical polymers used for tissue engineering. Colloids Surf., B 2007, 60 (2), 137–157. 10.1016/j.colsurfb.2007.06.019. 17683921

[ref22] Liang Y.; Jordahl J. H.; Ding H.; Deng X.; Lahann J. Uniform coating of microparticles using CVD polymerization. Chem. Vap. Deposition 2015, 21 (10–11–12), 288–293. 10.1002/cvde.201507197.

[ref23] Kumar R.; Welle A.; Becker F.; Kopyeva I.; Lahann J. Substrate-independent micropatterning of polymer brushes based on photolytic deactivation of chemical vapor deposition based surface-initiated atom-transfer radical polymerization initiator films. ACS Appl. Mater. Interfaces 2018, 10 (38), 31965–31976. 10.1021/acsami.8b11525. 30180547

[ref24] Hagenhoff B. High resolution surface analysis by TOF-SIMS. Microchim. Acta 2000, 132 (2), 259–271. 10.1007/s006040050019.

[ref25] Belu A. M.; Graham D. J.; Castner D. G. Time-of-flight secondary ion mass spectrometry: techniques and applications for the characterization of biomaterial surfaces. Biomaterials 2003, 24 (21), 3635–3653. 10.1016/S0142-9612(03)00159-5. 12818535

[ref26] Ko Y.; Ratner B.; Hoffman A. Characterization of hydrophilic—hydrophobic polymeric surfaces by contact angle measurements. J. Colloid Interface Sci. 1981, 82 (1), 25–37. 10.1016/0021-9797(81)90120-X.

[ref27] Koenig M.; Kumar R.; Hussal C.; Trouillet V.; Barner L.; Lahann J. pH-Responsive Aminomethyl Functionalized Poly (p-xylylene) Coatings by Chemical Vapor Deposition Polymerization. Macromol. Chem. PhyS 2017, 218 (9), 1600521 10.1002/macp.201600521.

[ref28] Wang D.; Russell T. P. Advances in atomic force microscopy for probing polymer structure and properties. Macromolecules 2018, 51 (1), 3–24. 10.1021/acs.macromol.7b01459.

[ref29] Inkson B. J. Scanning electron microscopy (SEM) and transmission electron microscopy (TEM) for materials characterization. In Materials Characterization Using Nondestructive Evaluation (NDE) Methods; Woodhead Publishing: Cambridge, 2016; pp 17–43 10.1016/B978-0-08-100040-3.00002-X.

[ref30] Cozzolino D. Benefits and limitations of infrared technologies in omics research and development of natural drugs and pharmaceutical products. Drug Dev. Res. 2012, 73 (8), 504–512. 10.1002/ddr.21043.

[ref31] Swanson K.; Trivedi S.; Lequieu J.; Swanson K.; Kondor R. Deep learning for automated classification and characterization of amorphous materials. Soft Matter 2020, 16 (2), 435–446. 10.1039/C9SM01903K. 31803878

[ref32] Bhatt P. M.; Malhan R. K.; Rajendran P.; Shah B. C.; Thakar S.; Yoon Y. J.; Gupta S. K. Image-based surface defect detection using deep learning: A review. J. Comput. Inf. Sci. Eng. 2021, 21 (4), 040801 10.1115/1.4049535.

[ref33] Singh S. A.; Desai K. A. Automated surface defect detection framework using machine vision and convolutional neural networks. J. Intell. Manuf. 2023, 34 (4), 1995–2011. 10.1007/s10845-021-01878-w.

[ref34] Jeihanipour A.; Lahann J. Deep-Learning-Assisted Stratification of Amyloid Beta Mutants Using Drying Droplet Patterns. Adv. Mater. 2022, 34 (24), 2110404 10.1002/adma.202110404. 35405768

[ref35] Vaez S.; Dadfar B.; Koenig M.; Franzreb M.; Lahann J. Deep Learning-Based Classification of Histone–DNA Interactions Using Drying Droplet Patterns. Small Sci. 2024, 4, 2400252 10.1002/smsc.202400252. 40213456 PMC11935254

[ref36] Dadfar B.; Vaez S.; Haret C.; Koenig M.; Mohammadi Hafshejani T.; Franzreb M.; Lahann J. Deep-Learning-Assisted Affinity Classification for Humoral Immunoprotein Complexes. Small Struct. 2024, 5, 2400204 10.1002/sstr.202400204.

[ref37] Mampallil D.; Eral H. B. A review on suppression and utilization of the coffee-ring effect. Adv. Colloid Interface Sci. 2018, 252, 38–54. 10.1016/j.cis.2017.12.008. 29310771

[ref38] Hu H.; Larson R. G. Marangoni effect reverses coffee-ring depositions. J. Phys. Chem. B 2006, 110 (14), 7090–7094. 10.1021/jp0609232. 16599468

[ref39] Sefiane K.; Duursma G.; Arif A. Patterns from dried drops as a characterisation and healthcare diagnosis technique, potential and challenges: A review. Adv. Colloid Interface Sci. 2021, 298, 102546 10.1016/j.cis.2021.102546. 34717206

[ref40] Hu Y.; Zhang X.; Qiu M.; Wei Y.; Zhou Q.; Huang D. From coffee ring to spherulites ring of poly (ethylene oxide) film from drying droplet. Appl. Surf. Sci. 2018, 434, 626–632. 10.1016/j.apsusc.2017.10.202.

[ref41] Lohani D.; Basavaraj M. G.; Satapathy D. K.; Sarkar S. Coupled effect of concentration, particle size and substrate morphology on the formation of coffee rings. Colloids Surf., A 2020, 589, 124387 10.1016/j.colsurfa.2019.124387.

[ref42] Carreón Y. J. P.; Gómez-López M. L.; Díaz-Hernández O.; Vazquez-Vergara P.; Moctezuma R. E.; Saniger J. M.; González-Gutiérrez J. Patterns in dried droplets to detect unfolded BSA. Sensors 2022, 22 (3), 1156 10.3390/s22031156. 35161907 PMC8839909

[ref43] Kim J.; Patra A.; Pal S.; Abbott N. L.; Lahann J. Emergent Properties, Functions, and Applications of Phane-Based Polymers. Adv. Funct. Mater. 2024, 34, 2315891 10.1002/adfm.202315891.

[ref44] Ünal Y.; Öztürk Ş.; Dudak M. N.; Ekici M. Comparison of current convolutional neural network architectures for classification of damaged and undamaged cars. In Advances in Deep Learning, Artificial Intelligence and Robotics: Proceedings of the 2nd International Conference on Deep Learning, Artificial Intelligence and Robotics,(ICDLAIR) 2020; Springer, 2022; pp 141–149 10.1007/978-3-030-85365-5_14.

[ref45] Sarkar S.; Kundu S. Protein (BSA) adsorption on hydrophilic and hydrophobic surfaces Mater. Today: Proc. 2023; Vol. 1 10.1016/j.matpr.2023.04.200.

[ref46] Whang S. E.; Roh Y.; Song H.; Lee J.-G. Data collection and quality challenges in deep learning: A data-centric ai perspective. VLDB J. 2023, 32 (4), 791–813. 10.1007/s00778-022-00775-9.

[ref47] Mohammadi Hafshejani T.; Zhong X.; Kim J.; Dadfar B.; Lahann J. Chemical and Topological Control of Surfaces Using Functional Parylene Coatings. Org. Mater. 2023, 5 (02), 98–111. 10.1055/s-0043-1761309.

[ref48] Deng X.; Lahann J. Orthogonal surface functionalization through bioactive vapor-based polymer coatings. J. Appl. Polym. Sci. 2014, 131 (14), 40315 10.1002/app.40315.

