# Supplementary material for: Deep Learning Based Surface Classification of Functionalized Polymer Coatings
Source: Langmuir. 2025 Apr 30;41(18):11272–83. doi: 10.1021/acs.langmuir.4c03971 (PMC12080330; doi:10.1021/acs.langmuir.4c03971)
Supplement: Supplementary file 1 — la4c03971_si_001.pdf [file la4c03971_si_001.pdf]

Supporting Information

Deep Learning Based Surface Classification of Functionalized Polymer Coatings

Safoura Vaez[1], *Diba Shahbazi*[1], *Meike Koenig*[1], *Matthias Franzreb*[1], *Joerg Lahann*[1,2*]

[1] Institute of Functional Interfaces (IFG), Karlsruhe Institute of Technology (KIT), Hermann-von-Helmholtz-Platz 1, 76344 Eggenstein-Leopoldshafen, Germany

[2] Biointerfaces Institute, Departments of Chemical Engineering, Materials Science and Engineering, and Biomedical Engineering, and the Macromolecular Science and Engineering Program, University of Michigan, Ann Arbor, MI 48109, USA

* *Corresponding author: Prof. J. Lahann; Email: lahann@umich.edu*

Number of pages: 15
Number of figures: 10
Number of tables: 3

Table of content

Figure S1) Analysis of the BSA-bicarbonate buffer stain using TOF-SIMS imaging. S2
Figure S2) Grad-CAM activation maps illustrating misclassified PLM images. S3
Figure S3) ToF-SIMS Survey spectra of A) negative and B) positive polarity measurements. S4-S5
Figure S4) IRRAS of various PPXs S6-S7
Figure S5) Classification performance of a pre-trained network on unseen PPX-Br patterns. S8
Figure S6) t-SNE visualizations of the feature space with perplexity values of 10, 50, and 100. ....S9
Figure S7) Grad-CAM activation maps. S10
Figure S8) Accuracy and loss curves for training and validation per epoch in surface recognition of PPX-coated glass wafers, in training the network conducted (i) with and (ii) without PPX-Br images. S11
Figure S9) Accuracy and loss curves per epoch for training and validation in network training on the effect of ionic strength in surface recognition. S12
Figure S10) Wettability of PPX-A and PPX-AM functioanl surfaces, measured by the size of dried BSA dissolvel in the buffer with and without sodium chloride. S13
Table S1) Precision, Recall, and F1-score derived from the confusion matrix for the network trained with 9 functionalized CVD coating surfaces.. ....S14
Table S2) Precision, Recall, and F1-score derived from the confusion matrix for the network trained with 8 functionalized CVD coating surfaces (excluding PPX-Br).. ....S14
Table S3) Precision, Recall, and F1-score derived from the confusion matrix for the network trained to evaluate the effect of ionic strength on functionalized surface classification... ....S15

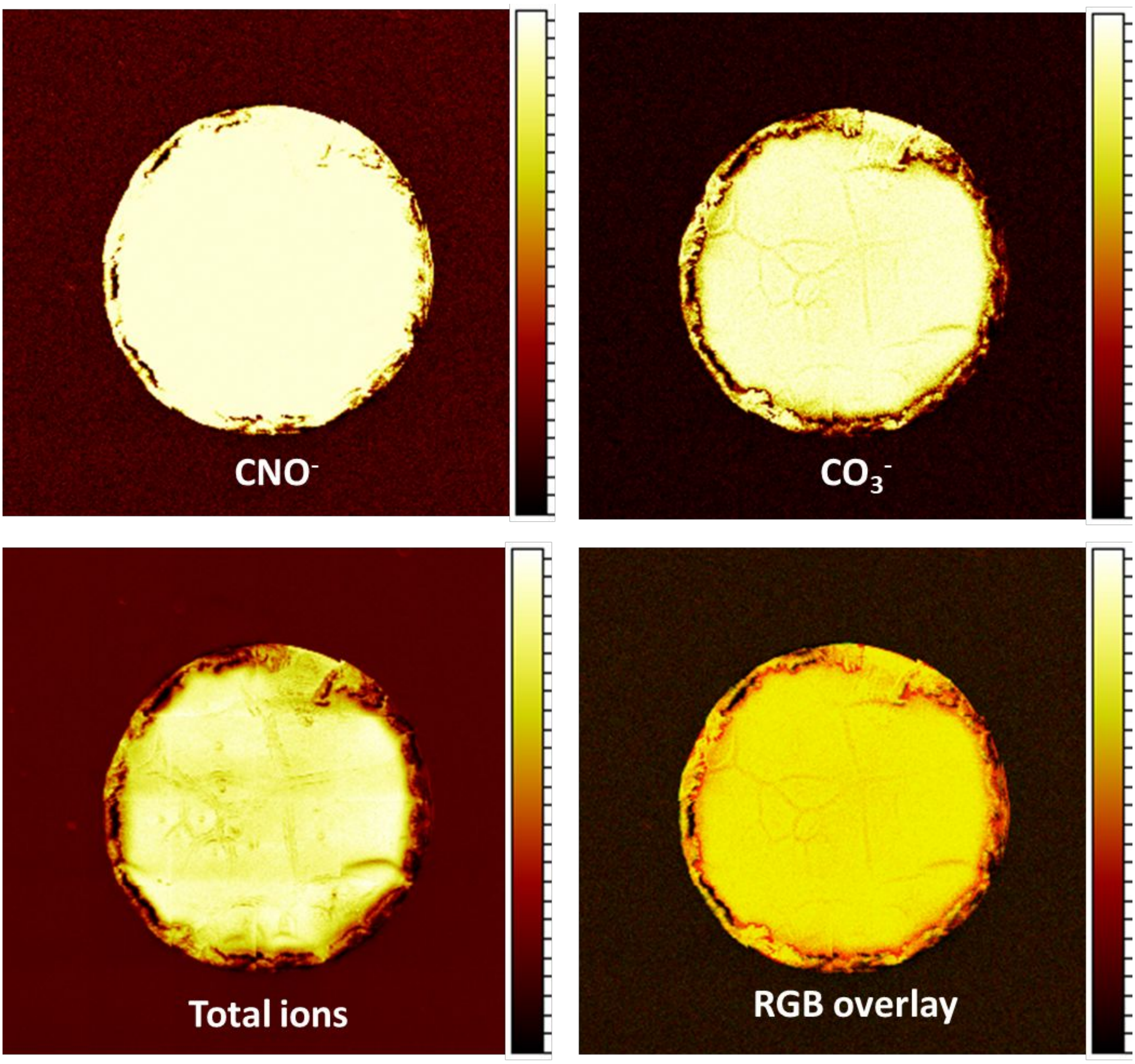

Figure S1) Analysis of the BSA-bicarbonate buffer stain using TOF-SIMS imaging. Brown-scale images of the BSA protein dissolved in bicarbonate buffer: CNO^- indicates the protein, while CO_3^- represents the carbonate buffer (scale bar for both 0-53 counts, for total ions 0-2850 counts). In the RGB overlay image, red indicates CNO^-, and green represents CO_3^-.

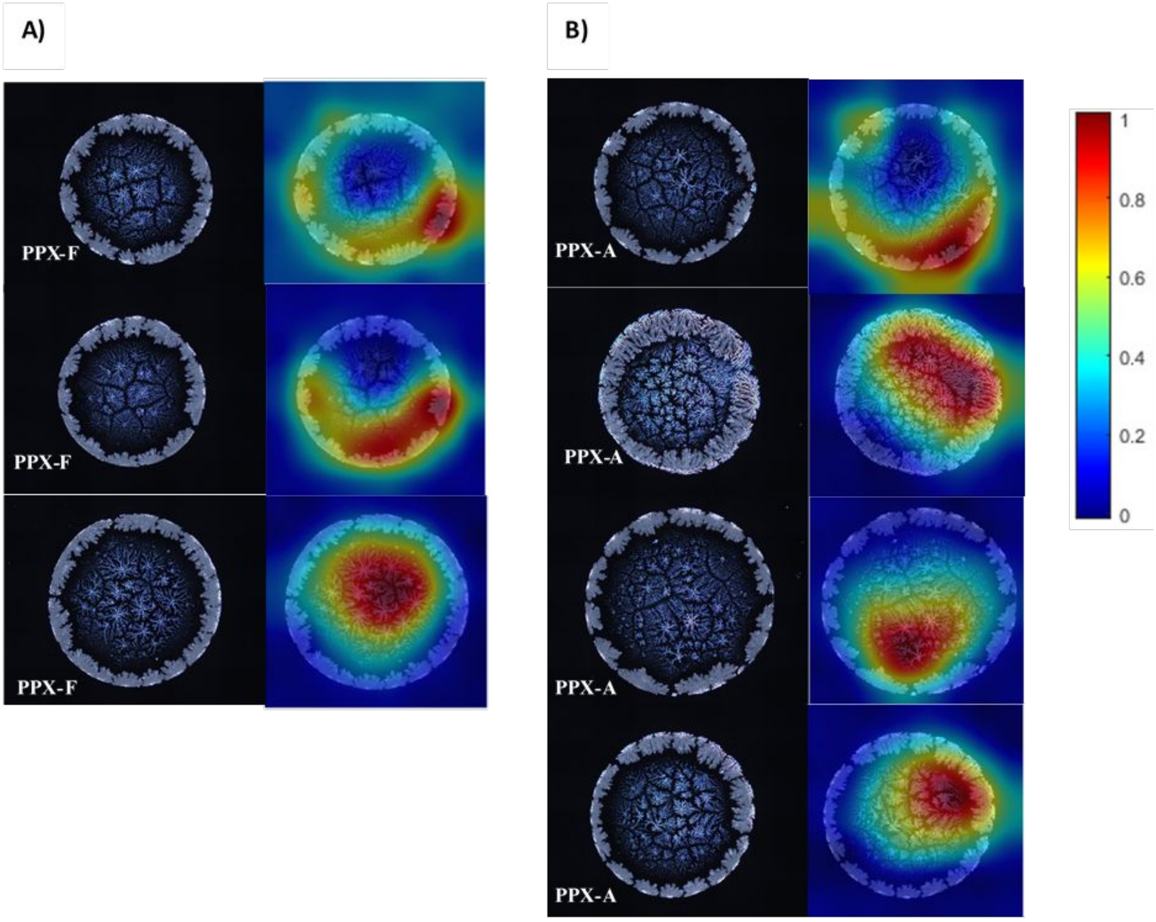

Figure S2) Grad-CAM activation maps illustrating misclassified PLM images: A) PPX-F as PPX-Alkyne; Three misclassifications occurred out of 80 unseen PLM images of BSA deposition patterns on PPX-F. B) PPX-A as PPX-OH; Four misclassifications occurred out of 80 unseen PLM images of BSA deposition patterns on PPX-A. Background interference, pattern-free spaces, and improper patterns collected from the edges of coated substrates (where the coating may have been insufficient) were the most causes of this misclassification.

A)

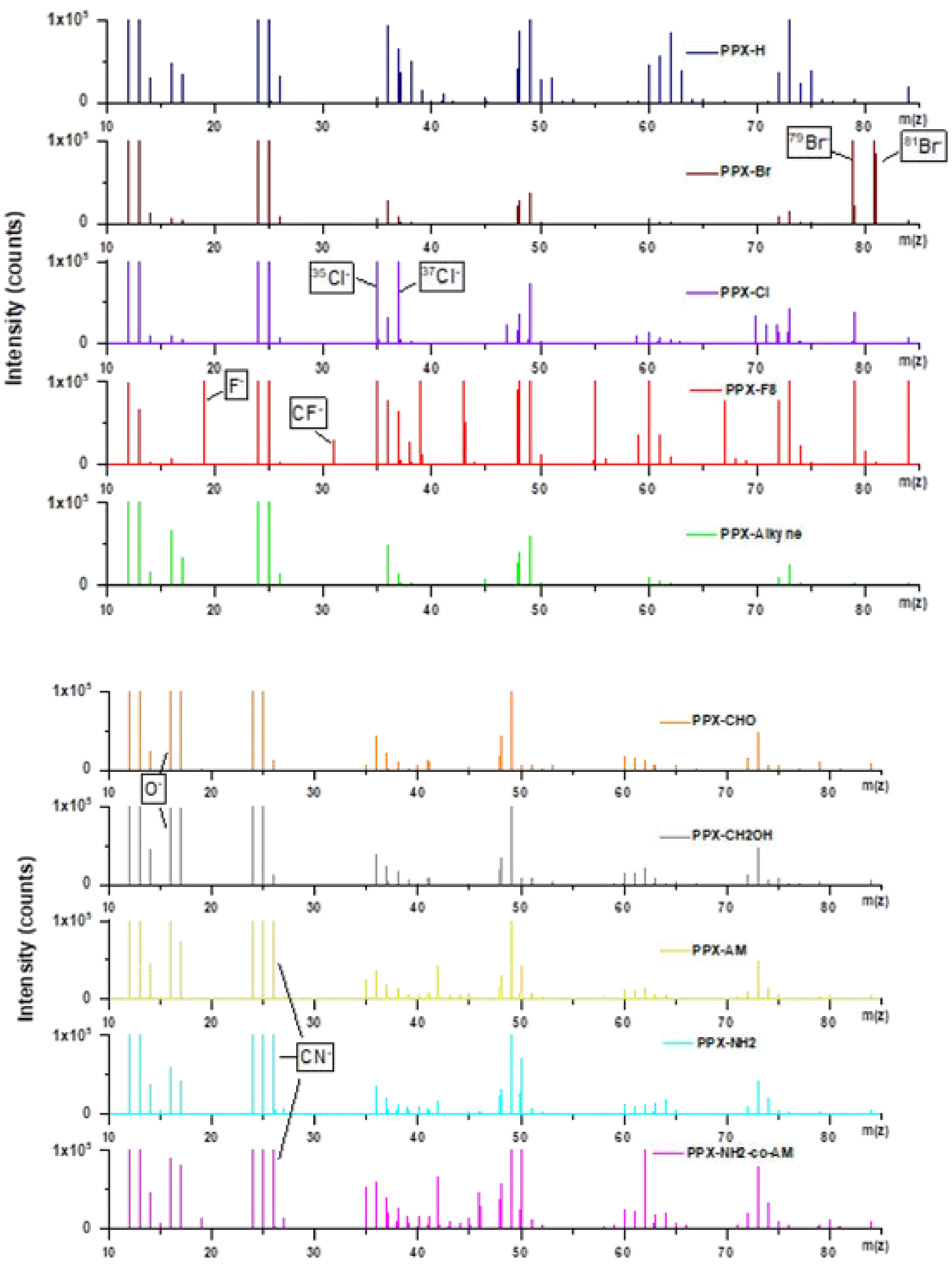
Intensity (counts)
PPX-H
PPX-Br
PPX-Cl
PPX-F8
PPX-Alkyne
PPX-CHO
PPX-CH2OH
PPX-AM
PPX-NH2
PPX-NH2-co-AM
$^{79}Br^-$
$^{81}Br^-$
$^{35}Cl^-$
$^{37}Cl^-$
F^-
CF^-
O^-
CN^-
1x10^5
0
m(z)
10
20
30
40
50
60
70
80

B)

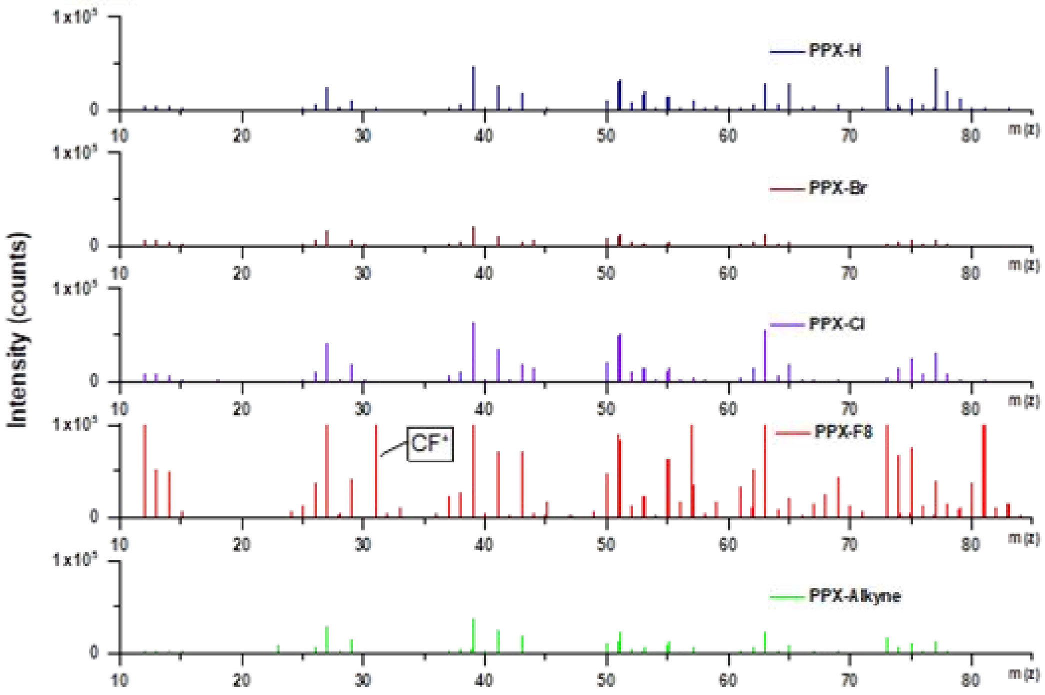

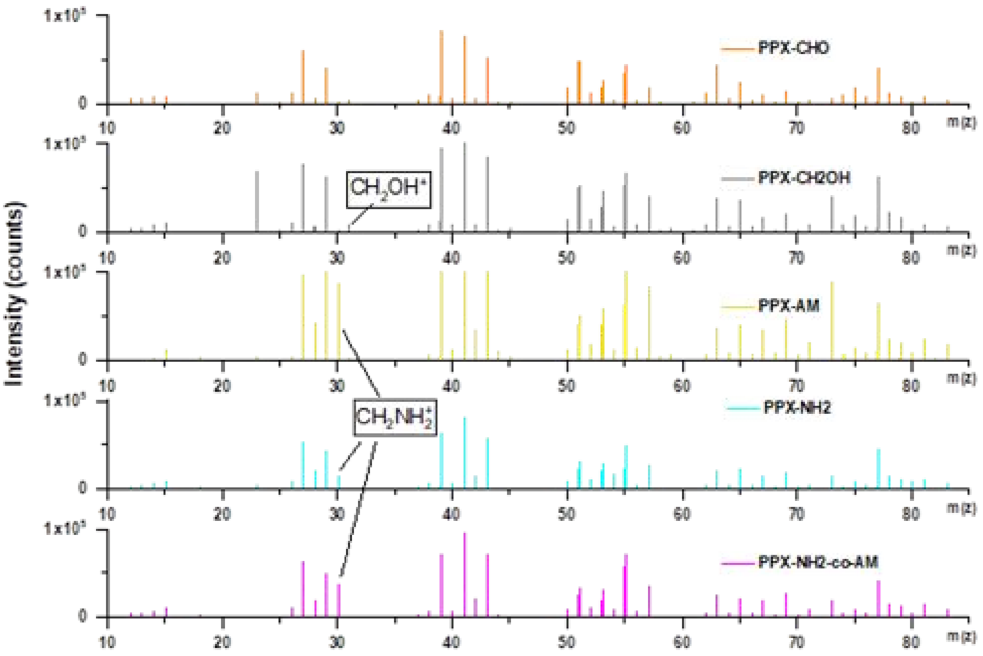

Figure S3) ToF-SIMS Survey spectra of A) negative and B) positive polarity measurements.

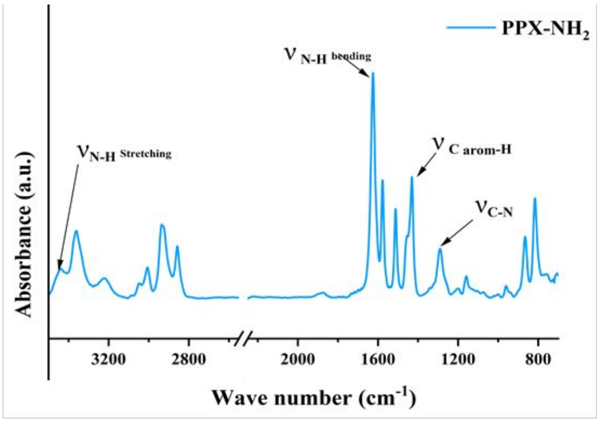
PPX-NH2
ν N-H bending
ν N-H Stretching
ν C arom-H
ν C-N
Absorbance (a.u.)
3200
2800
2000
1600
1200
800
Wave number (cm-1)

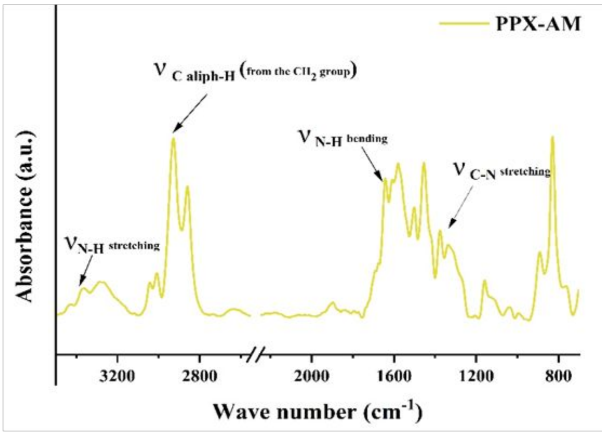
PPX-AM
ν C aliph-H (from the CH2 group)
ν N-H bending
ν C-N stretching
ν N-H stretching
Absorbance (a.u.)
3200
2800
2000
1600
1200
800
Wave number (cm-1)

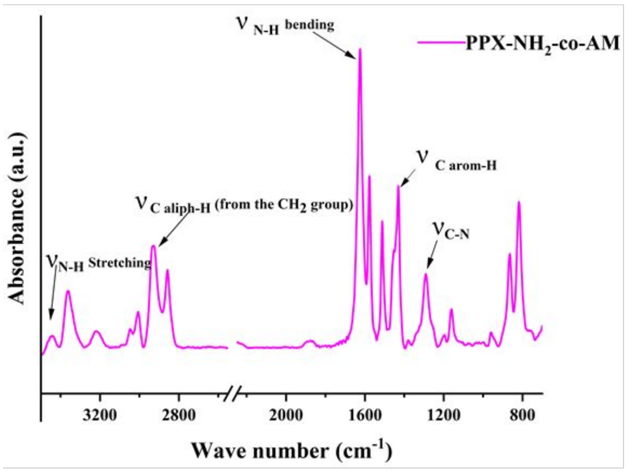
ν N-H bending
PPX-NH2-co-AM
ν C arom-H
ν C aliph-H (from the CH2 group)
ν C-N
ν N-H Stretching
Absorbance (a.u.)
3200
2800
2000
1600
1200
800
Wave number (cm-1)

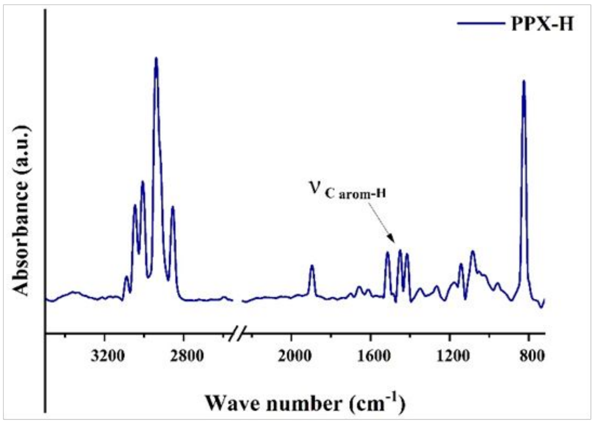
PPX-H
ν C arom-H
Absorbance (a.u.)
3200
2800
2000
1600
1200
800
Wave number (cm-1)

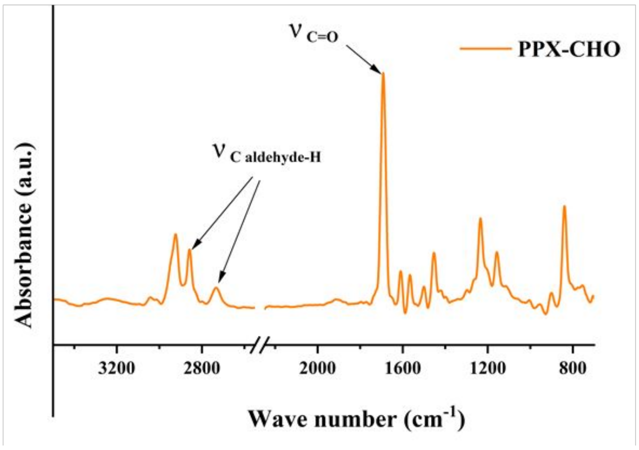
ν C=O
PPX-CHO
ν C aldehyde-H
Absorbance (a.u.)
3200
2800
2000
1600
1200
800
Wave number (cm-1)

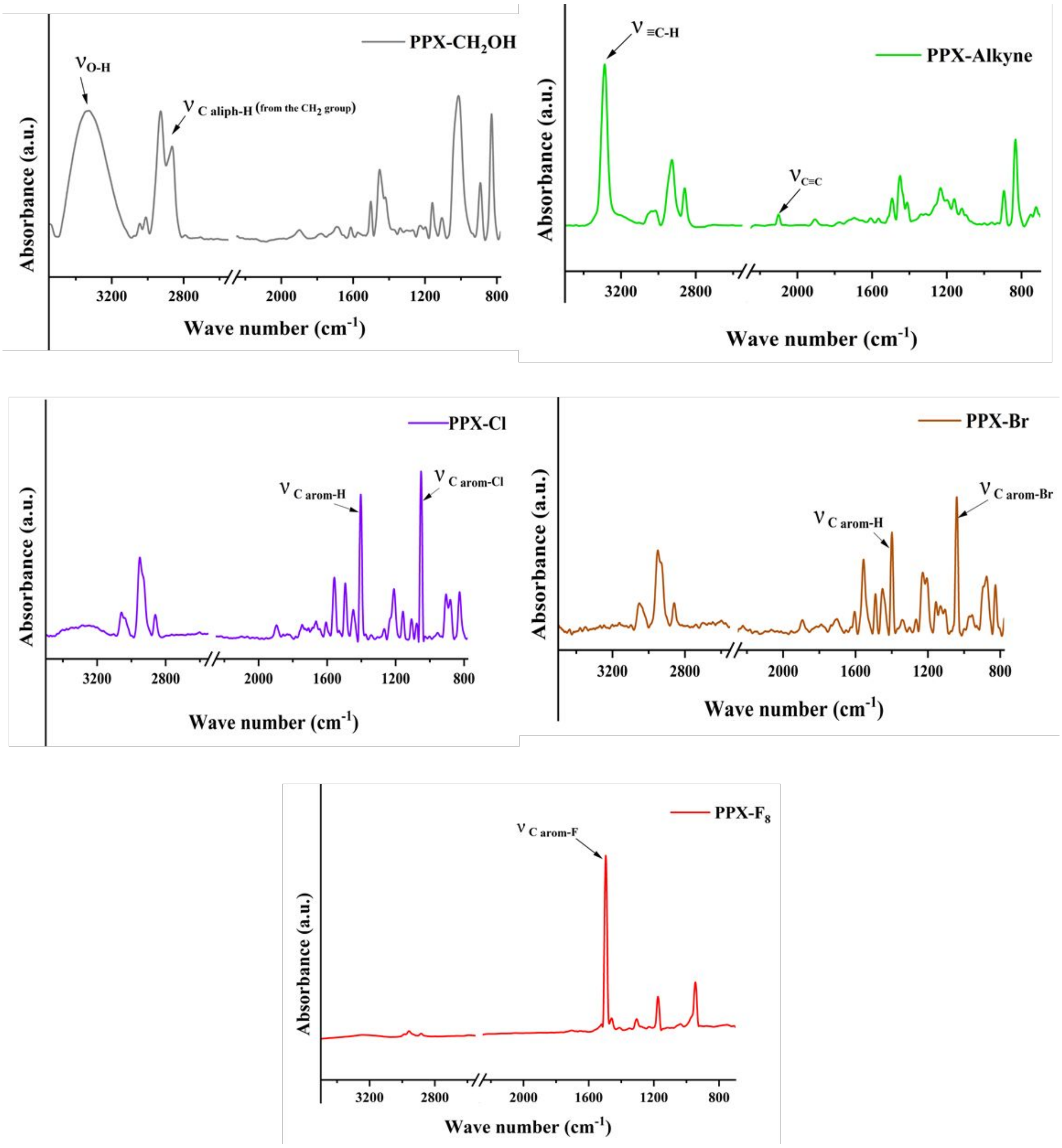

Figure S4) IRRAS of various PPXs

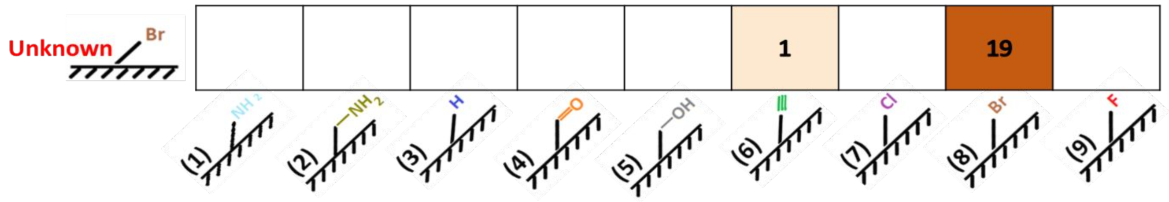

Figure S5) Classification performance of a pre-trained network on unseen PPX-Br patterns.

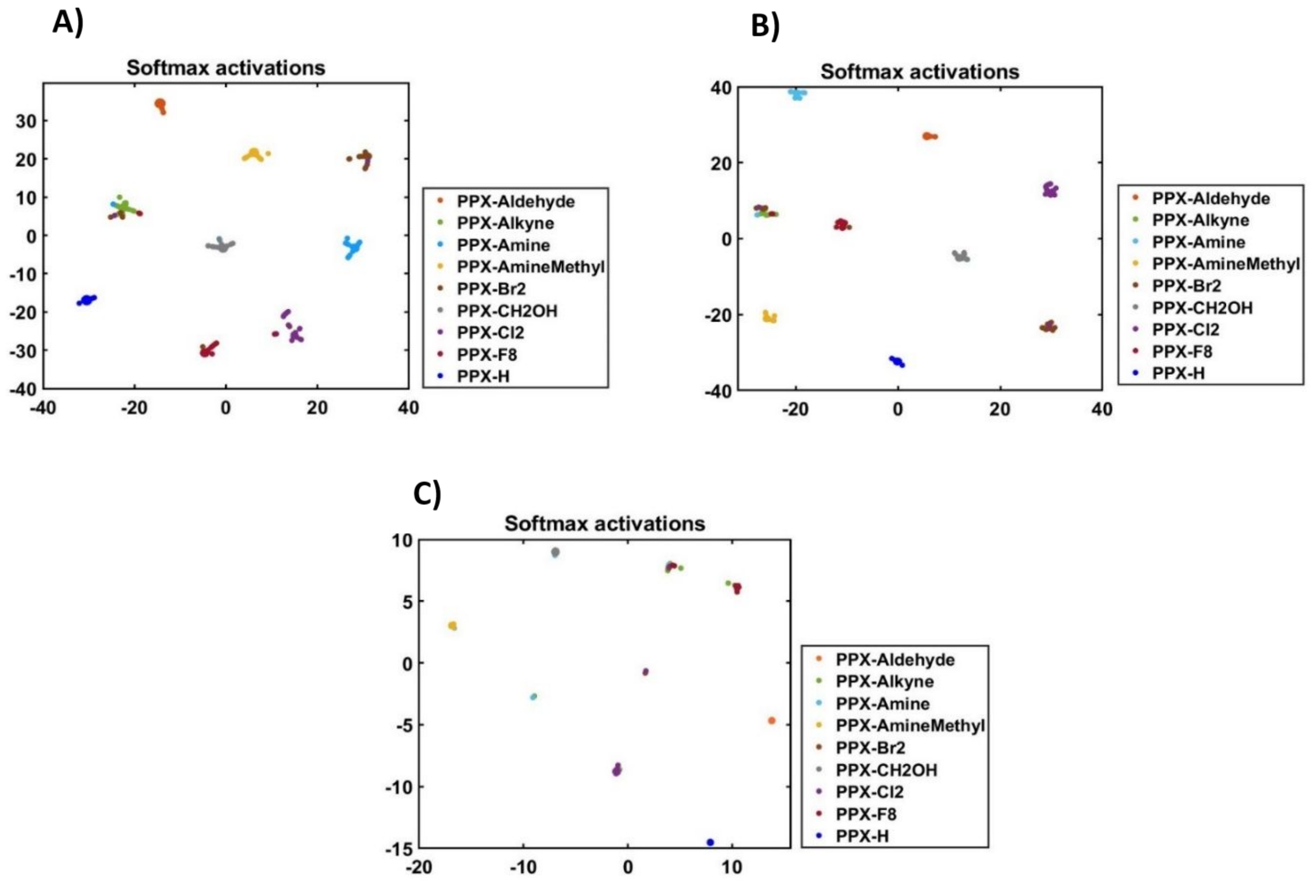

Figure S6) t-SNE visualizations of the feature space with perplexity values of 10, 50, and 100. These values were chosen to examine the robustness of clustering across both local (low perplexity) and global (high perplexity) relationships. The consistent clustering observed across all perplexity values indicates robust feature representations by the network.

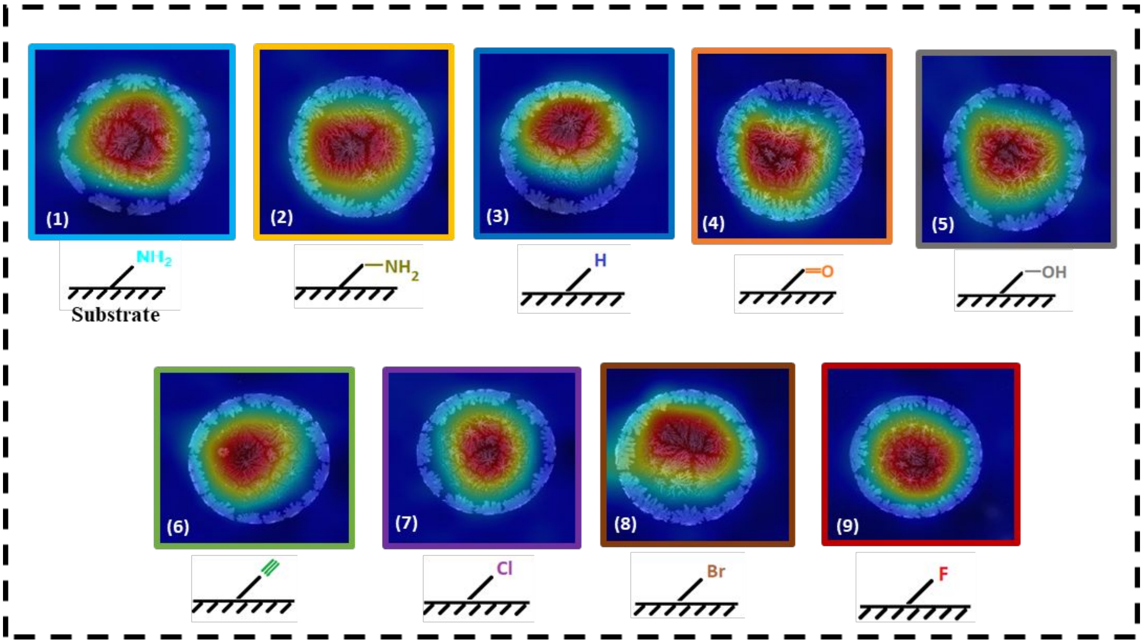

Figure S7) Grad-CAM activation maps

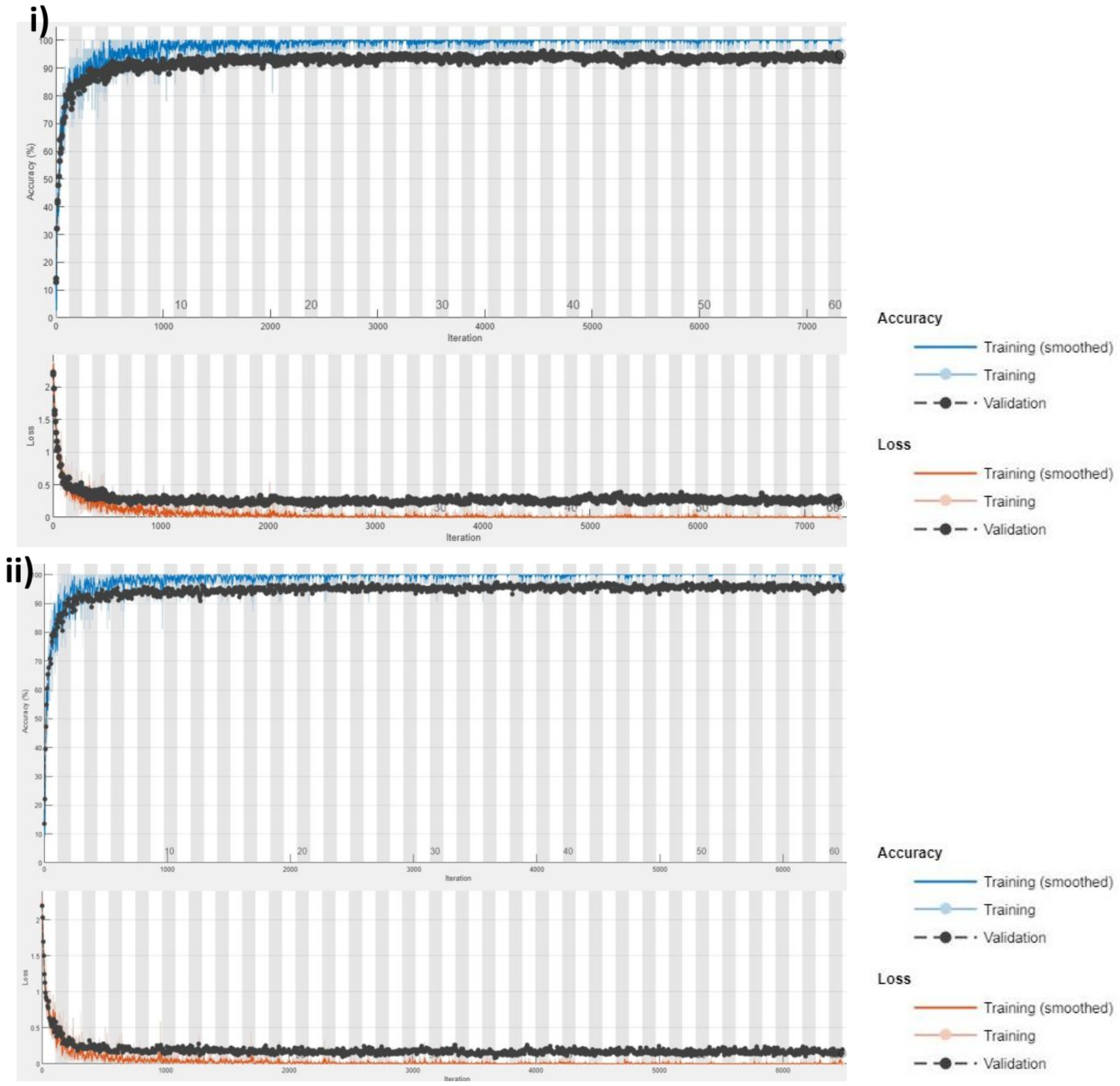

Figure S8) Accuracy and loss curves for training and validation per epoch in surface recognition of PPX-coated glass wafers, in training the network conducted (i) with and (ii) without PPX-Br images.

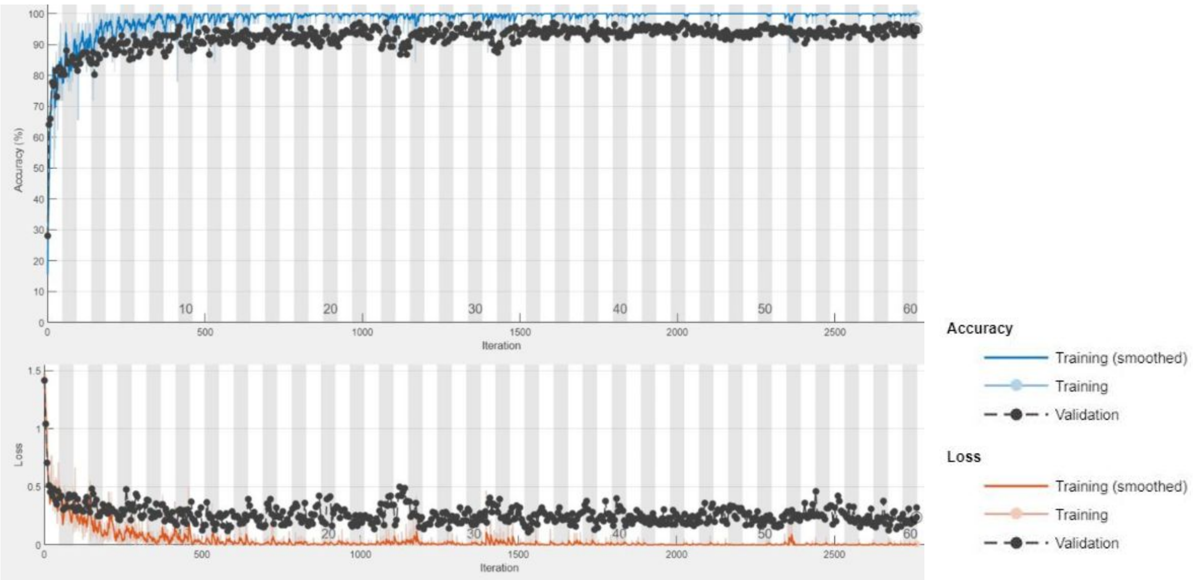

Figure S9) Accuracy and loss curves per epoch for training and validation in network training on the effect of ionic strength in surface recognition.

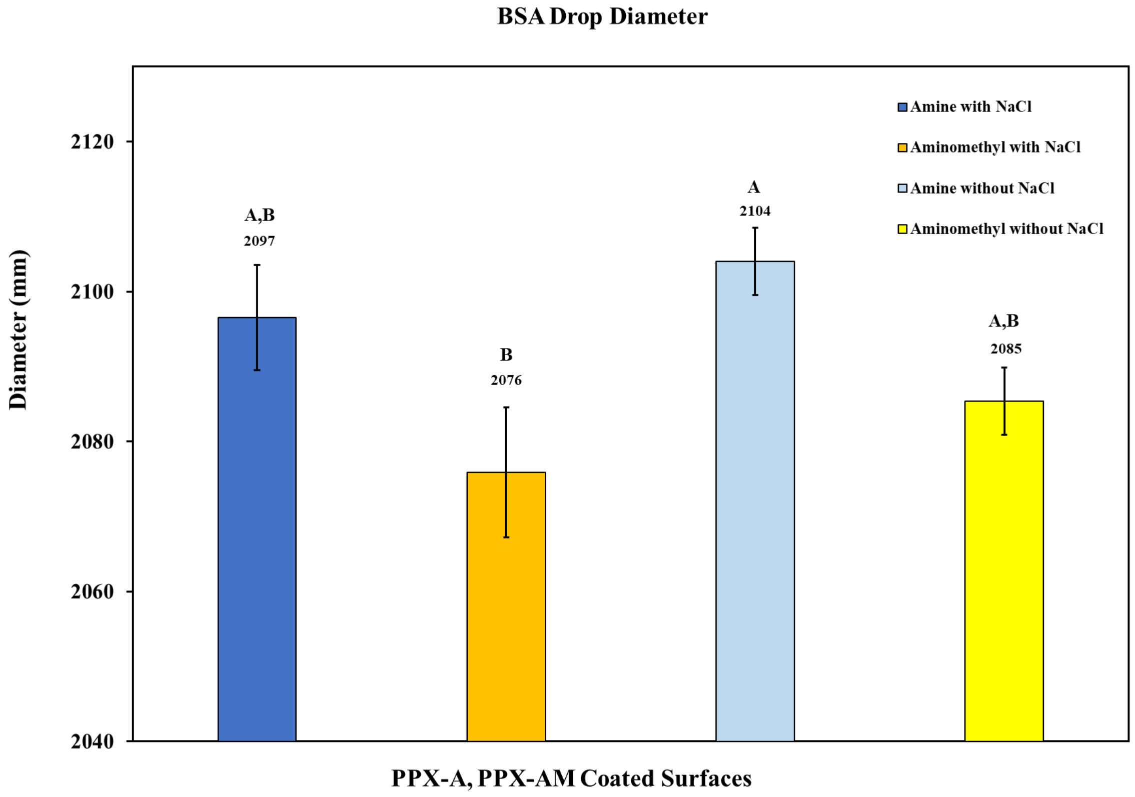

Figure S10) Wettability of PPX-A and PPX-AM functioanl surfaces, measured by the size of dried BSA dissolvel in the buffer with and without sodium chloride . The diameter of 150 randomly selected stains was measured for each group. Error bars show standard Error (SE). Means with the same letters are not significantly different (Tukey-adjusted comparisons). The Tukey technique was applied for the determination of the significant difference with $p < 0.05$.

Recall (sensitivity)for class i:

$$Recall = \frac{True\ positive\ (TP)}{True\ positive\ (TP) + False\ negative\ (FN)}$$

Precision for class i:

$$Precision = \frac{True\ positive\ (TP)}{True\ positive\ (TP) + False\ positive\ (Fp)}$$

F1-score for class i:

$$F1\_score = 2 * \frac{Precision * Recall}{precision + Recall}$$

Table S1: Precision, Recall, and F1-score derived from the confusion matrix for the network trained with 9 functionalized CVD coating surfaces.

class	TP	FN	FP	precision	Recall	F1-Score
1 (A)	74	6	1	0.9867	0.9250	0.9548
2 (AM)	80	0	1	0.9877	1.0000	0.9938
3(H)	80	0	0	1.0000	1.0000	1.0000
4 (Ald)	80	0	0	1.0000	1.0000	1.0000
5 (OH)	80	0	4	0.9524	1.0000	0.9756
6 (Alk)	73	7	10	0.8795	0.9125	0.8957
7 (Cl)	78	2	7	0.9176	0.9750	0.9455
8 (Br)	67	13	4	0.9437	0.8375	0.8874
9 (F)	77	3	4	0.9506	0.9625	0.9565

We compute the average for all classes:

- Precision: 0.9576
- Recall: 0.9569
- F1-score: 0.9566

Table S2: Precision, Recall, and F1-score derived from the confusion matrix for the network trained with 8 functionalized CVD coating surfaces (excluding PPX-Br).

class	TP	FN	FP	precision	Recall	F1-Score
1 (A)	77	3	0	1.0000	0.9625	0.9809
2 (AM)	79	1	1	0.9875	0.9875	0.9875
3(H)	80	0	0	1.0000	1.0000	1.0000
4 (Ald)	80	0	2	0.9756	1.0000	0.9877
5 (OH)	80	0	1	0.9877	1.0000	0.9938
6 (Alk)	78	2	7	0.9176	0.9750	0.9455
7 (Cl)	79	1	3	0.9634	0.9875	0.9753
9 (F)	72	8	1	0.9863	0.9000	0.9412

- Precision: 0.9773
- Recall: 0.9766
- F1-score: 0.9765

Table S3: Precision, Recall, and F1-score derived from the confusion matrix for the network trained to evaluate the effect of ionic strength on functionalized surface classification.

class	TP	FN	FP	precision	Recall	F1-Score
PPX-A	78	2	0	1.0000	0.9750	0.9873
PPX-MA	80	0	2	0.9756	1.0000	0.9877
PPX-A	69	11	1	0.9857	0.8625	0.9200
PPX-MA	79	1	11	0.8778	0.9875	0.9294

We compute the average for all classes:

- Precision: 0.9598
- Recall: 0.9563
- F1-score: 0.9561
